# Limited Sexual Reproduction and Quick Turnover in the Population Genetic Structure of *Phytophthora infestans* in Fujian, China

**DOI:** 10.1038/srep10094

**Published:** 2015-05-13

**Authors:** Wen Zhu, Li-Na Yang, E-Jiao Wu, Chun-Fang Qin, Li-Ping Shang, Zong-Hua Wang, Jiasui Zhan

**Affiliations:** 1 Fujian Key Lab of Plant Virology, Institute of Plant Virology, Fujian Agriculture and Forestry University, Fuzhou, Fujian, P. R. China; 2 Key Lab for Biopesticide and Chemical Biology, Ministry of Education, Fujian Agriculture and Forestry University, Fuzhou, Fujian, P. R. China

## Abstract

The mating system plays an important role in the spatiotemporal dynamics of pathogen populations through both its direct and indirect impact on the generation and distribution of genetic variation. Here, we used a combination of microsatellite and phenotypic markers to investigate the spatiotemporal distribution of genetic variation in *Phytophthora infestans* isolates collected from Fujian, China and to determine the role of sexual reproduction in the dynamics. Although the pathogen populations in this region were dominated by self-fertile genotypes, sexual reproduction only occurred occasionally and its contributions to the population genetic structure of *P. infestans* and epidemics of late blight in the region were limited. Only 49 genotypes were detected among the 534 isolates assayed and the pathogen populations displayed significant heterozygosity excess. Hierarchical analysis revealed that 21.42% of genetic variation was attributed to the difference among sampling years while only 4.45% was attributed to the difference among locations, suggesting temporal factors play a more important role in the population genetic dynamics of *P. infestans* than spatial factors in this region. We propose that clonal reproduction, combined with founder effects and long distance dispersal of sporangia, is responsible for the observed pattern of spatiotemporal dynamics in *P. infestans*.

The mating system is one of the main evolutionary factors driving the spatial and temporal dynamics of genetic variation in pathogen populations. In nature, pathogens may vary in mating systems spatially and temporally and the ability to engage in various mating systems may be a ‘bet-hedging’ strategy that enables pathogens to adapt better to a range of biotic and abiotic environments^1^.

Mating systems affect the amount and distribution of genetic variation within pathogen populations. Clonal or inbreeding populations are expected to exhibit low genetic variation and a significant degree of non-random association among unlinked loci. In contrast, outcrossing populations are expected to display high genetic variation and random associations among neutral loci due to the re-assortment of unlinked genes during meiosis^2^. Mating systems can also affect the extent of genetic drift and gene flow in pathogen populations. Inbreeding increases the extent of genetic drift in pathogen populations, resulting from reduced effective population size, because the reproductive success of offspring is no longer dependent on the two independent alleles per locus^3^. Inbreeding depression may also increase the contribution of gene flow to the reproductive success of local populations if recombinants between immigrants and residents were competitively superior^4^,^5^ while random mating generally increases effective population size of a pathogen^6^,^7^. Due to repeating cycles of extinction and re-colonization of local populations associated with host dynamics, chemical applications and changes in cultural practices and/or extensive trade of plant materials and agricultural commodities locally and globally^8^,^9^, the extents of genetic drift and gene flow in the pathogen populations are expected to be high in modern agriculture.

The oomycete *P. infestans,* the cause of late blight disease, is a notorious plant pathogen responsible for the Irish potato famine in the 1840s, and is still the most devastating disease of potato and tomato worldwide^10^,^11^, particularly in areas experiencing moderate temperature and high humidity. The pathogen can affect all parts of a potato crop, including leaves, stems and tubers. Under favorable climatic conditions, the entire potato crop can be destroyed within a few days. The annual losses caused by late blight are estimated to exceed ~$6.7 billion worldwide^12^. Since 1993, China has become the world’s leading potato production country^13^,^14^, accounting for 26.3% and 22.2% of global acreage and yield, respectively^15^. Late blight is the main factor affecting the sustainability of potato production in this region^16^.

Despite the existence of self-fertile genotypes^17^,^18^,^19^,^20^,^21^, *P. infestans* is considered to be a heterothallic oomycete in which the occurrence of sexual reproduction requires the presence of two opposite mating types (A1 and A2)^22^. Before the 1980s, the global population of *P. infestans* outside of Mexico reproduced asexually and was dominated by a single clonal lineage of A1 mating type termed HERB-1 and then US-1^23^. The introduction of the A2 mating type to regions outside of Mexico^24^ and emergence of self-fertile pathotypes in recent years in many parts of the world make sexual reproduction possible in *P. infestans* globally^17^,^18^,^19^,^20^,^21^. Sexual reproduction is expected to increase the genotypic diversity within *P. infestans* populations due to the continuous rearrangement of existing alleles or generation of new alleles through intragenic recombination. Regular sexual reproduction in heterothallism can also homogenize the frequencies of A1 and A2 mating types attributed to balancing selection^9^. However, recent surveys indicate that *P. infestans* still reproduces primarily in a clonal manner and sexual reproduction is rare^25^,^26^,^27^,^28^ in most parts of the world with an exception in North Europe^29^.

Although exhibiting low genetic variation overall, temporal analysis of its population dynamics indicates that *P. infestans* has high evolutionary potential in nature^30^. New genotypes can be generated through mutation such as transposable events and other genetic mechanisms and fittest genotypes can spread out to large geographical areas. For example, the clonal lineage HERB-1 which persisted for over 50 years and the US-1 dominating the pre-1970s collection^23^ are rarely identified now and have been replaced by other lineages with higher aggressiveness and fungicide resistance^31^,^32^. A single *P. infestans* genotype called *13*_A2 was first detected in the Netherlands in 2004 at a very low frequency^33^. A few years later, this genotype was detected in many European countries including the United Kingdom^33^,^34^,^35^. In China, one of the dominant clonal lineages is genetically similar to *13*_A2^16^.

Fujian is a mountainous province located in Southeastern China and is classed as a winter potato production zone. Potato in this region always rotates with rice or other non-*Solanum* crops and its production primarily relies on seed tubers imported from other parts of the country. This combination of landscape structure and production system may have important impacts on the population genetic dynamics and evolutionary trajectory of *P. infestans*. To understand the population genetic structure of *P. infestans* in Fujian and infer potential evolutionary mechanisms responsible for the structure, we performed a three-year molecular and phenotypic surveys in the regions. Therefore. The main objectives of this study are: i) to estimate genetic and phenotypic variation of *P. infestans*; ii) to investigate spatiotemporal population genetic dynamics of *P. infestans*; and iii) to determine whether sexual reproduction and genetic drift are responsible for the observed spatiotemporal genotypic diversity in this population of *P. infestans*.

## Results

### Genotypic and gene diversity

**Figure 1:**
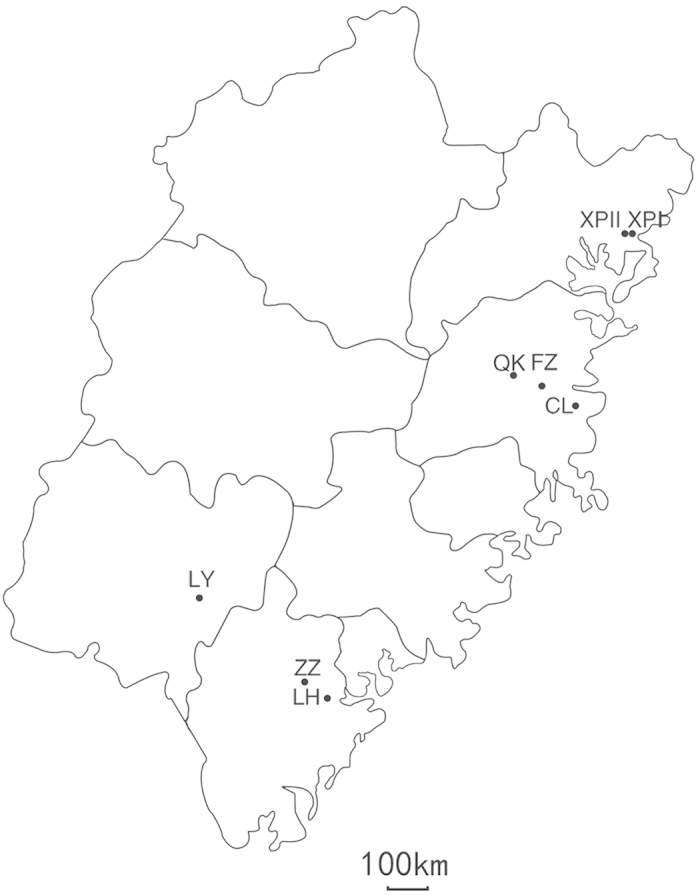
Map of Fujian province showing the locations of the eight populations of *P. infestans*. Adobe Illustrator Artwork 17.0 software was used to create the map.

**Figure 2:**
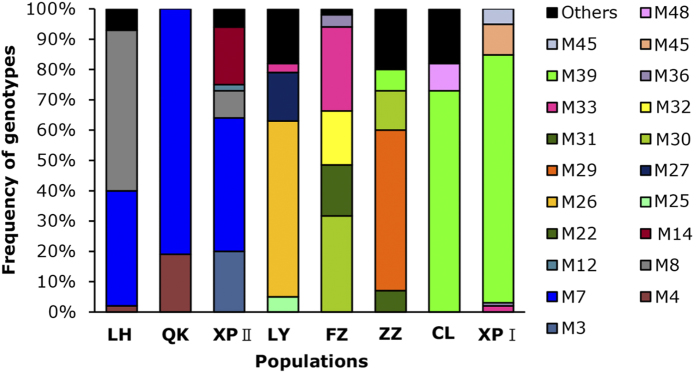
Geographic distribution of the main multilocus genotypes in the *P. infestans* populations from Fujian, China.

**Figure 3:**
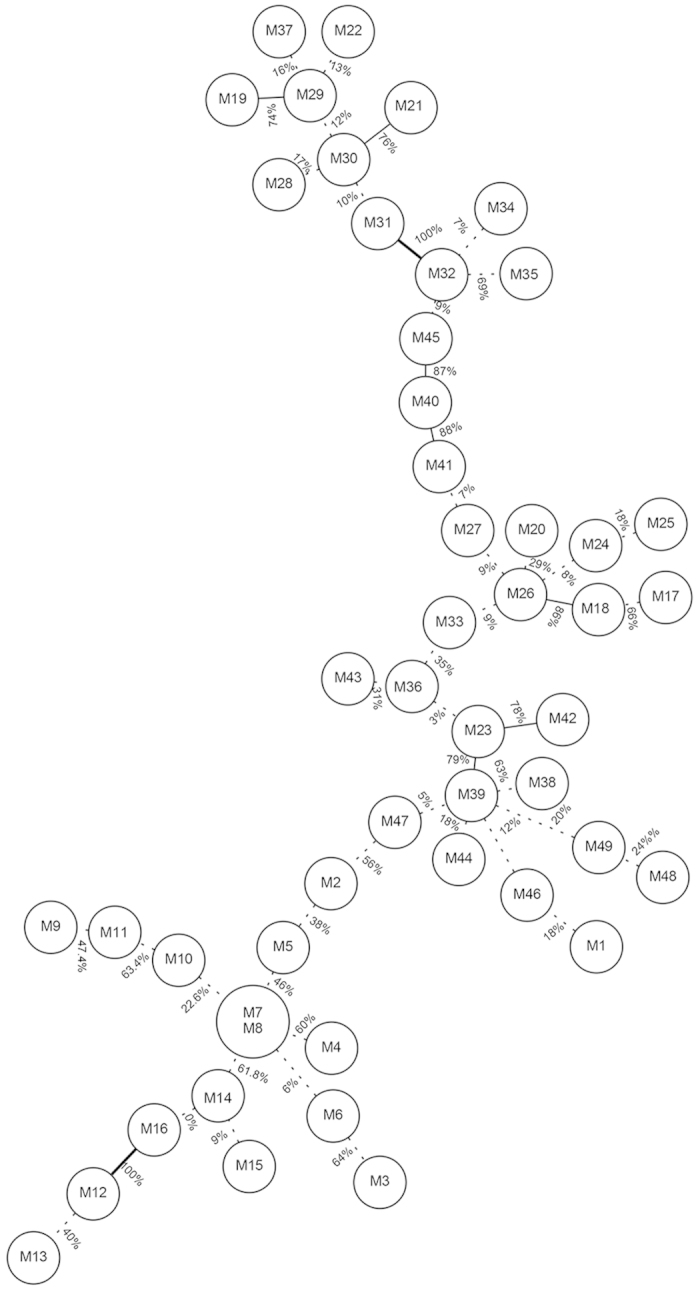
Phylogenetic tree of the 49 *P. infestans* genotypes reconstructed by Minimum spanning using SSR data. The digits in the edges represent percentage bootstrap values obtained after 500 iterations. A solid bold edge indicates a bootstrap value over 90%; solid line indicates a value over 70% and a dotted line indicates a value below 70%. The mean bootstrap percent is 39 ± 4.5.

Potato leaves infected with *P. infestans* were collected at random from 8 commercial fields located in Fujian Province in the early stage of epidemics during 2010–2012 (Table 1; Fig. 1). Among 534 *P. infestans* isolates amplified with eight SSR markers (Supplementary Table 1), 49 distinct genotypes were detected. Twenty-nine of the genotypes were detected only once and seven genotypes (M4, M7, M8, M30, M33, M36, and M39) were shared in populations sampled from different locations or years (Fig. 2). Among the seven shared genotypes, five (M7, M8, M30, M36 and M39) are likely to be the ancestral strains (Fig. 3). M7 was detected in three populations (LH, QK and XPII) sampled from 2012 and M33 was detected in three populations sampled from three different years (LY, XPI and FZ). No identical genotypes were detected between XPI and XPII (Fig. 2). M7 was the most common genotype, accounting for 81.3%, 44.1% and 37.8% of QK, XPII and LH populations, respectively (Fig. 2). M39 was the second most common genotype, accounting for 81.3%, 72.7% and 6.7% of XPI, CL and ZZ populations, respectively. In general, genotype richness (R) was low in all populations, with the highest genotype richness observed in ZZ (R = 0.31) and the lowest in QK (R = 0.01) (Table 1), and each population was dominated by 1-2 genotypes (Fig. 2). A total of 30 alleles were detected over eight microsatellite loci, ranging from two alleles in locus Pi33 to five alleles in loci G11 and Pi16 (Table 2). The number of alleles observed among populations as an average across eight loci ranged from 1.88 to 2.63 (Table 1). Expected heterozygosity (*He*) was similar across all populations, ranging from 0.37 in LH to 0.46 in ZZ (Table 1).

### Mating type variation and multilocus associations

Of the 534 isolates tested, 197 (36.89%) formed oospores with the A2 reference strain and were considered to be A1 mating type. The remaining 337 isolates (63.11%) formed oospores by themselves as well as with the A1 and A2 reference strains and were considered as self-fertile. No A2 mating type was detected in any of the populations from Fujian Province (Table 3). Multilocus association analysis indicates that all populations but CL deviated significantly from the hypothesis of linkage equilibrium (Table 1). The association index in QK could not be estimated due to its low genotype diversity (2 genotypes, Fig. 2).

### Population differentiation

**Figure 4:**
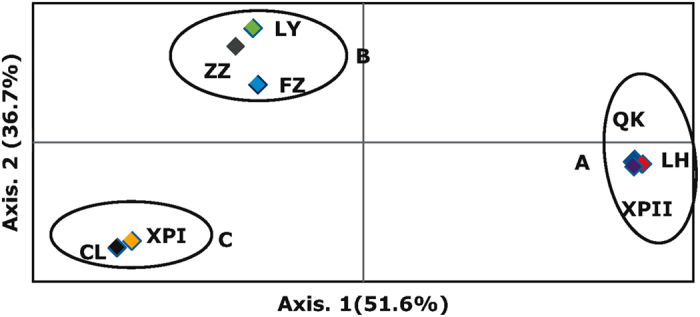
Principal component analysis (PCA) of the *P. infestans* populations from Fujian by a non-hierarchical Bayesian cluster using eight SSR markers. Three clusters were revealed from the analysis with 3 (LH, QK and XPII), 3 (LY, ZZ and FZ) and 2 (XPI and CL) populations in Cluster A, B and C, respectively.

**Figure 5:**
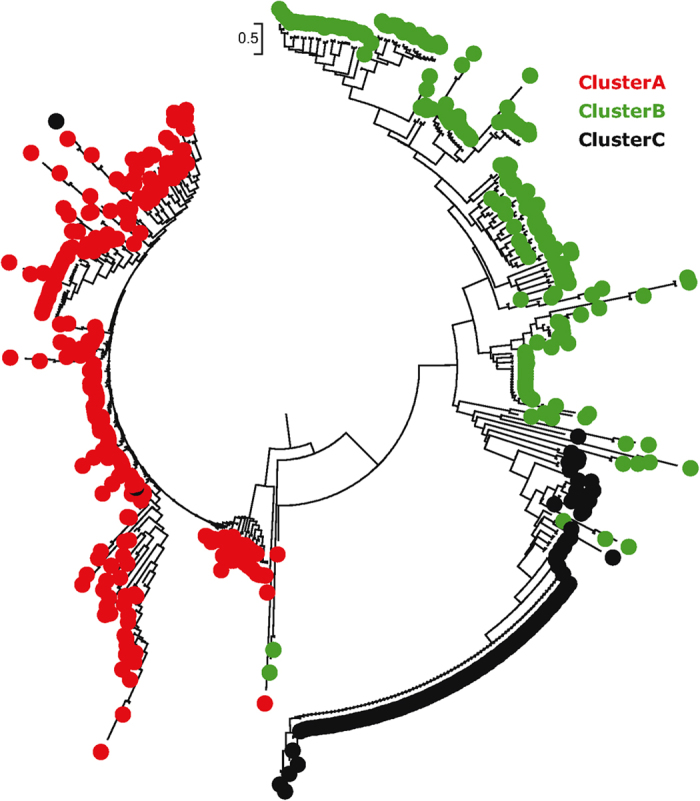
Neighbor joining tree showing the phylogenetic relatedness among the 543 *P. infestans* isolates sampled from Fujian, China. Nei’s genetic distance was calculated using GENALEX 6.5 and the phylogenetic tree was reconstructed in Mega 5.

PCA revealed that the 1^st^ and 2^nd^ components accounted for 51.60% and 36.71% of total genetic variability, respectively (Fig. 4). Based on the first two dimensions of the PCA, populations from Fujian were grouped into three clusters (A, B and C), comprising 3 (LH, QK and XPII), 3 (LY, ZZ and FZ) and 2 (XPI and CL) populations, respectively (Fig. 4). Isolates in cluster A were all self-fertile while isolates in Cluster C were all A1 mating type (Table 3). Cluster B was the admixture of both A1 and self-fertile isolates. With few exceptions, isolates from the same PCA clusters were also grouped together phylogenetically (Fig. 5).

**Figure 6:**
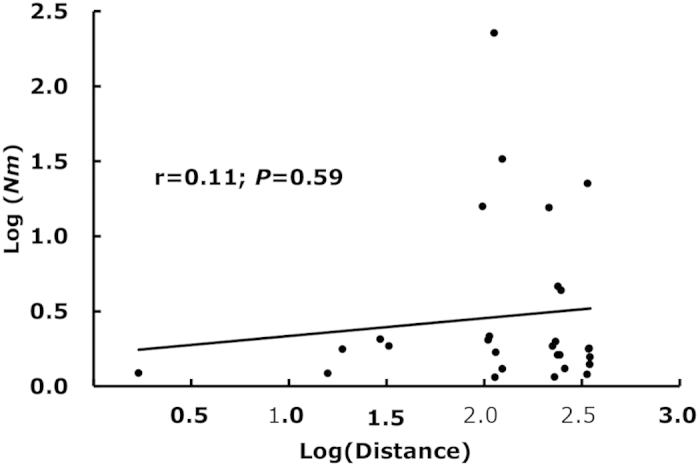
Correlation between geographic distance and the extent of gene flow in the *P. infestans* populations from Fujian, China.

Significant difference in allele frequency was found in all eight SSR loci among the eight populations and between XPI and XPII (Table 2). Pairwise *F*_ST_ ranged from 0.001 to 0.310 (Table 4). All except two pairs (XPI-CL and QK-XPII) of *F*_ST_ were significantly different from zero. The correlation coefficient between log (physical distance) and log (*N*m) among all 8 geographic populations was 0.11, which was not significant (*P *= 0.59, Fig. 6).

The analysis of molecular variance (AMOVA) performed on the eight populations indicated that 21.42% and 4.45% of the genetic variation was attributed to variations among years and among locations within year, respectively (Table 5). At the cluster level, AMOVA indicated that 23.98, 1.91 and 74.12% of the total variation was attributed to among clusters, among populations within clusters and among individual isolates within each population, respectively. All of the variances from the AMOVA were highly significant (Table 5).

## Discussion

Since its first report outside Mexico, the possible center of origin of *P. infestans*^36^, the A2 mating type has been observed in many parts of the world^24^. Though tremendous variations exist across these studies, the frequency of the A2 mating type was generally lower than A1 mating type^27^,^34^ with a few exceptions. In Fujian, the A2 mating was detected in the *P. infestans* populations sampled before 2007^37^, but it seems that it was absent in this area after 2010. We did not find any A2 mating type isolates in the 534 isolates assayed. With this sample size, there is a 99% chance of detecting an A2 mating type if it has a frequency of 0.5% or higher^8^. Less than the expected frequency (50%) in many studies suggests the A2 mating type might have an overall lower fitness than its A1 counterpart. If the A2 mating type is linked to low fungicide resistance, lower pathogenicity or other maladaptive characters, negative selection for these ecological traits would gradually reduce its frequency in populations. Similarly, in the wheat pathogen *Zymoseptoria tritici* (*Mycosphaerella graminicola*), Zhan and colleagues assayed the pathogenicity of nearly 200 isolates sampled from four continents and found a consistent pattern of higher aggressiveness in one mating type over the other^38^.

We found that more than two-third of the isolates in the current study were self-fertile. It has been reported that environmental factors such as the types of media used to culture *P. infestan*s play an important role in the determination of its mating types^39^. Oatmeal, tomato juice and V8-juice can induce the conversion of A2 mating type isolates to a self-fertile state. In our study, we used rye B agar, which cannot induce A2 mating type isolates to produce oospores by itself, to culture and determine the mating types of *P. infestans*. The self-fertile isolates detected in Gansu province, China can also produce oospores when they were inoculated onto potato leaves^19^. This result suggests a recent increase of self-fertile genotypes in Fujian, consistent with results from other studies in China^19^ and other parts of the world^18^,^20^ though we do not know the genetic, evolutionary and/or ecological causes of this increase.

Self-fertile isolates can not only produce oospores by themselves but also with the A1 and A2 isolates. It is thus expected that *P. infestans* populations from Fujian would have a moderate to high genetic variation, as reported in North and West Europe^29^,^40^. However, the genetic variation in the Fujian populations is low. We only detected 49 genotypes among the 543 isolates and many populations were dominated by 1-2 genotypes, suggesting that sexual reproduction (including selfing by self-fertile isolates and/or outcrossing between self-fertile and A1 isolates) might occur in the *P. infestans* populations, but its contributions to the population genetic structure of the pathogen and epidemics of late light in the region is limited.

Hierarchical analysis revealed that more than 20% of genetic variation was attributed to the difference in sampling years, while only ~5% was attributed to the difference in locations, suggesting temporal factors make more important contributions to the population genetic dynamics of *P. infestans* than spatial factors in Fujian. This pattern of spatiotemporal population dynamics in *P. infestans* was also evident by comparing the population genetic structure of the pathogen from the same location but different years. XPI and XPII were sampled from two nearby fields planted with the same potato cultivar at one year interval but high genetic differentiation in SSR frequency, no identical genotypes, and different mating types were found in the two populations.

Genetic drift in terms of founder effect combined with long dispersal ability of *P. infestans* may account for the observed pattern of spatiotemporal population structure. Agriculture is characterized by a recurrent expansion (growing season) and contraction (harvest season) of both host and pathogen populations^8^,^9^. With each cycle of contraction, the size of the pathogen population collapses sharply and new populations in the next cycle of expansion are usually derived from a small number of genotypes randomly surviving after the last contraction. This leads to a dramatic change in the population genetic structure of pathogens from year to year, particularly for pathogens with small effective population sizes. Due to limited number (Table 1) and skewed distribution (Fig. 2) of genotypes, the effective population size of *P. infestans* in Fujian is expected to be small and its genetic drift caused by the seasonal population contraction is expected to be severe in this region. Fujian is located in a subtropical temperature zone with long and hot summers. Its air temperature in July and August can reach 40 °C or higher and last for several hours in a day. At the night time, the air temperature in this season usually is not less than 25 °C. Such environmental condition further escalates genetic drift in *P. infestans*, causing rapid changes in population genetic structure over time. However, due to the potential for long distance movement of *P. infestans* sporangia^41^, particularly in areas such as Fujian which experiences many typhoons each year and the short generation time of the pathogen^10^, the progenies of newly established founder populations may travel hundreds of kilometers away from their original location during a single season in a stepping stone event^42^,^43^, reducing the difference of genetic structure among local populations. Indeed, the geographical distances between any pairs of populations involved in this study are less than 350 km (Fig. 1) and there are no physical barriers, such as high mountains, constraining the movement of sporangia between populations. However, genetic drift and gene flow in this case may never reach an equilibrium, leading to an high estimate of population subdivision (Table 4).

Founder effects generated by infected seed tubers may also contribute to the observed pattern of population genetic dynamics of *P. infestans*. Most potato seed tubers in Fujian are imported from other parts of China. The seed tubers are usually purchased from different producers and their quality is regulated poorly, bringing different sets of pathogen populations from different parts of the country each year. Natural populations usually display a negative correlation between the extent of gene flow and geographic distance, defined as isolation by distance^44^. The finding of no isolation by distance in the current study is consistent with the hypothesis that human-mediated gene flow through infected seed tubers may play an important role in the spatiotemporal population genetic structure of *P. infestans* in Fujian.

Though dominated by self-fertile genotypes, our analyses indicate that *P. infestans* in this region mainly reproduces asexually and the contribution of sexual reproduction to the population genetic structure of the pathogen is limited. This epidemic mode of reproduction that combines many cycles of asexual propagation with fewer cycles of sexual reproduction allows *P. infestans* to preserve favorable allele combinations that are well adapted to existing hosts and environments while retaining its ability to generate novel allele combinations when hosts and environments are changing, breaking the effect of Muller’s ratchet^45^. Mixed modes of reproduction, combined with extensive human-mediated gene flow, facilitate the generation and spread of novel virulence factors and the breakdown of host resistance. Though it is difficult to change its reproductive system, gene flow (particularly human mediated gene flow) in *P. infestans* can be regulated through good agricultural practices, strict quarantine procedures and public awareness of biosafety and biosecurity. To effectively control late blight, it is important to curb human-mediated gene flow by using clean seed and plant materials in addition to the dynamic use of resistance genes and chemicals^9^.

## Materials and methods

### *P. infestans* collection and isolation

Potato leaves infected with *P. infestans* were collected at random from 8 commercial fields located in Fujian province in the early stage of epidemics during 2010–2012 (Fig. 1). For all collections, infected leaves were sampled at more than 2-m intervals from different parts of a field, carefully dried with soft tissue paper, placed in sealed sandwich bags immediately and transported to the laboratory within 24 hours for isolation. Only one infected leaf was sampled from each potato plant and each infected leaf was packed in a separate sandwich bag. XPI and XPII were collected from different fields separated by ~2 km in the same village.

To isolate the pathogen, plant materials were rinsed with sterilized distilled water. A piece of tissue was cut from the margin of leaf lesion and placed abaxial side up on 2.0% water agar for 20–30 hours. A single piece of mycelium was hooked out from sporulating lesion aseptically using an inoculating needle, transferred to a rye B agar^46^ plate supplemented with Ampicillin (100 mg/L) and Rifampin (10 mg/L) and maintained at 18 °C in the dark for seven days to form colony. To purify the pathogen, a new piece of mycelium was pieced off the colonies, transferred onto a fresh rye B agar plate and incubated at 18 °C in the dark for another 7–10 days.

### DNA extraction

Mycelia (~100 mg) were obtained by culturing *P. infestans* isolates on rye B agar at 18 °C in the dark for 15 days, transferred into sterile, 2 mL centrifuge tubes and lyophilized with a vacuum freeze dryer (Alpha1-2, Christ). The lyophilized mycelia were ground to powders with a mixer mill (MM400, Retsch). Total DNA was extracted using Plant gDNA Miniprep Kit (GD 2611, Biomiga, China) according to the manufacturer’s instructions. The genomic DNA was suspended in 200 μL ultrapure water and stored at −20 °C until use.

### SSR analysis

Genomic DNA from each of the *P. infestans* isolates was amplified with eight SSR markers (Pi02, Pi04, Pi4B, PiG11, Pi16, Pi33, Pi56 and Pi89). Pi4B and PiG11 markers were developed by Knapova and Gisi^47^ and Pi02, Pi04, Pi16, Pi33, Pi56 and Pi89 markers were developed by Lees and her colleagues^48^. Primers of the SSR markers were labeled with one of four fluorescent dyes: FAM for Pi56 and Pi89, ROX for PiG11 and Pi04, HEX for Pi02, and Pi16, and TAMRA for Pi33 and Pi4B.

PCR amplifications were performed in a 25 μL volume containing 1 μL of *P. infestans* genomic DNA (~20 ng), 12.5 μL of 2× PCR Buffer Mix (TransGen Biotech Co., Ltd., Beijing, China), 1.0 μM each of forward and reverse primers in a 2720 thermal cycler (Applied Biosystems, USA) with the following conditions: initiated with a cycle of 2 min at 94 °C, followed by 35 cycles of 30 s at 94 °C, 25 s at 56 °C (for PiG11), 57 °C (for pi56), 58 °C (for Pi02, Pi04, Pi16, Pi33 and Pi4B) or 61 °C (for Pi89) and 60 s at 72 °C, and finished with an elongation cycle of 5 min at 72 °C. PCR products from the eight markers were mixed into two groups with PiG11, Pi56, Pi02 and Pi33 as one group and Pi89, Pi4B, Pi04 and pi16 as another based on fluorescent dyes used to label the primers and expected sizes of amplifications. Mixed PCR products, were loaded into 96-well plates and sent to Ruiboxingke Biotechn. Co. LTD. (Beijing) to determine fragment sizes using an ABI3730XL automated DNA sequencer (Applied Biosystems, Foster, California) in which a DNA size ladder was included in each of the samples. Alleles were assigned using GeneMapper software version 3.7 (Applied Biosystems).

### Mating type determination

Mating type was determined as described previously^37^. Briefly, each *P. infestans* isolate was paired with an A1 tester (US970001), an A2 (US940480) or grown alone on rye B agar plates. The two mating type testers were kindly provided by Prof. Qinghe Chen in Fujian Academy of Agricultural Sciences, Fuzhou, China. After inoculation at 18 °C in the dark for 12–15 days when the colonies from testers and tested isolates were fused, a piece of tissue was picked off from the intersected edge and observed microscopically to determine the production of oospores. The isolates forming oospores with A2 tester were designated as A1 mating type. Similarly, the isolates forming oospores when paired with the A1 tester were designated as A2 mating type. The isolates forming oospores with both testers and alone were designated as self-fertile. Mating type determination was replicated twice for each isolate.

### Data analysis

In analyzing the variation in microsatellite loci, most *P*. *infestans* isolates showed a maximum of two alleles per locus as expected for a diploid organism. However, for several cases, three alleles were detected in one locus. Loci with three alleles were modified to resemble diploid loci by assigning specific allele sizes and a binary representation of the presence or absence of specific alleles as described previously^34^. Multilocus genotype (MLG) for each isolate was formed by joining alleles at each SSR locus in the same order. Number of MLGs, was determined using the software GENCLONE 2.0^49^,^50^. Genotype diversity was calculated for each population using the genotypic richness R = (number of different MLGs - 1) / (total number of isolates - 1). Expected heterozygousity, observed heterozygousity and mean number of alleles (*N*_*a*_) were calculated by POPGEN v3.2.

Pairwise population differentiation (*F*_ST_) and gene flow (*N*_m_) were estimated using GENALEX 6.5. The physical distance between pair of populations was estimated using the geographic coordinates of locations from which the populations were sampled and isolation by distance was evaluated by estimating the association between the extent of gene flow and logarithm of physical distance (km) in the populations^44^. Genetic variation was partitioned by the analysis of molecular variance (AMOVA) using ARLEQUIN 3.5. Multilocus association in each population was tested using the software LIAN3.0. The test evaluates the null hypothesis of linkage equilibrium among loci by calculating the standardized index of association I_A_. All population genetic parameters were estimated with clonal uncorrected data. Principal component analysis (PCA) was performed with GENALEX 6.5 to produce map depicting the distribution of genetic diversity across geographic space. Genetic distances between isolates was calculated using GENALEX6.5 and the phylogenetic trees of the 534 isolates and 49 genotypes were reconstructed by a neighbor joining approach using MEGA5 and MST goldsoftware programme as described by Salipante and Hall^51^, respectively.

## Author Contributions

W.Z. and L.N.Y. collected pathogen isolates, generated and analyzed the data and wrote the paper; E.J.W., C.F.Q., L.P.S. and Z.H.W. collected pathogen isolates and generated the data; and J.Z. conceived and designed the experiments, analyzed the data and wrote the manuscript.

## Additional Information

**How to cite this article**: Zhu, W. *et al*. Limited Sexual Reproduction and Quick Turnover in the Population Genetic Structure of *Phytophthora infestans* in Fujian, China. *Sci. Rep.* **5**, 10094; doi: 10.1038/srep10094 (2015).

## Supplementary Material

Supplementary Information

## Figures and Tables

**Table 1:** Genetic diversity parameters of the eight *P. infestans* populations and each Cluster.

**Population/cluster**	**Abbreviation**	**Year**	**N**	***Na***	**M**	**R**	***He***	***I***_**A**_^**S**^
Longhai	LH	2012	45	2.4	6	0.11	0.37	0.06**
Qingkou	QK	2012	75	1.9	2	0.01	0.4	*--*
Xiapu-II	XPII	2012	127	2.6	12	0.09	0.39	0.41**
Longyan	LY	2012	101	2.3	7	0.06	0.4	0.08**
Fuzhou	FZ	2010	38	2.5	11	0.27	0.45	0.10**
Zhangzhou	ZZ	2010	30	2.4	10	0.31	0.46	0.20**
Changle	CL	2010	22	2.1	6	0.24	0.42	0.03
Xiapu-I	XPI	2011	96	2.1	5	0.04	0.46	0.07**
Cluster A			247	3	16	0.06	0.39	0.03**
Cluster B			169	3.1	26	0.15	0.43	0.09**
Cluster C			118	2.3	9	0.07	0.46	0.08^**^

^**:^Significance at P <0.001; Sample size (N), average number of alleles detected (*N*_*a*_), number of multilocus genotype (M), genotypic richness (R), expected heterozygousity (*H*_*e*_) and index of multilocus association (*I*_A_^S^).

**Table 2:** Allelic Frequencies of eight microsatellite loci in the eight populations of *P. infestans* sampled from Fujian, China.

**Locus**	**Allele**	**LH**	**QK**	**XPII**	**LY**	**FZ**	**ZZ**	**XPI**	**CL**	**Χ**^**2**^**-test**
G11	154	0.000	0.000	0.000	0.000	0.026	0.000	0.000	0.000	1076.8**
	156	0.011	0.000	0.000	0.500	0.447	0.500	0.500	0.523	
	158	0.011	0.000	0.008	0.500	0.526	0.500	0.500	0.477	
	160	0.489	0.500	0.496	0.000	0.000	0.000	0.000	0.000	
	162	0.489	0.500	0.496	0.000	0.000	0.000	0.000	0.000	
PI02	154	0.000	0.000	0.000	0.243	0.000	0.433	0.000	0.000	369.8**
	160	0.000	0.000	0.106	0.094	0.026	0.017	0.000	0.000	
	162	0.500	0.500	0.500	0.500	0.513	0.500	0.500	0.500	
	164	0.500	0.500	0.394	0.163	0.461	0.050	0.500	0.500	
PI04	166	0.000	0.000	0.000	0.000	0.013	0.000	0.000	0.000	39.3*
	168	0.500	0.500	0.500	0.500	0.487	0.500	0.500	0.500	
	170	0.000	0.000	0.000	0.000	0.026	0.000	0.000	0.000	
	172	0.500	0.500	0.500	0.500	0.474	0.500	0.500	0.500	
PI16	172	0.000	0.000	0.000	0.000	0.039	0.000	0.000	0.045	376.1**
	174	0.211	0.500	0.441	0.158	0.355	0.400	0.500	0.432	
	176	0.789	0.500	0.551	0.658	0.605	0.600	0.500	0.523	
	178	0.000	0.000	0.004	0.000	0.000	0.000	0.000	0.000	
	180	0.000	0.000	0.004	0.183	0.000	0.000	0.000	0.000	
PI33	203	1.000	1.000	0.996	0.975	1.000	0.933	0.536	0.500	387.6**
	206	0.000	0.000	0.004	0.025	0.000	0.067	0.464	0.500	
PI56	173	0.500	0.500	0.484	0.500	0.513	0.433	0.068	0.045	146.5**
	175	0.500	0.500	0.512	0.500	0.487	0.567	0.932	0.955	
	183	0.000	0.000	0.004	0.000	0.000	0.000	0.000	0.000	
PI89	175	0.011	0.093	0.000	0.000	0.000	0.000	0.000	0.000	388.2**
	177	0.956	0.907	0.996	0.500	0.526	0.550	0.500	0.500	
	179	0.033	0.000	0.004	0.500	0.474	0.450	0.500	0.500	
PI4B	207	0.500	0.500	0.508	0.000	0.000	0.050	0.484	0.523	1249.0**
	213	0.000	0.000	0.000	0.000	0.487	0.000	0.000	0.000	
	215	0.489	0.500	0.492	1.000	0.513	0.900	0.031	0.000	
	219	0.011	0.000	0.000	0.000	0.000	0.050	0.484	0.477	

^*^Significance at *P *< 0.01.

^**^Significance at *P *< 0.001.

**Table 3:** Mating type frequencies in the eight field populations and three Bayesian clusters o*f P. infestans* from Fujian, China

**Population/cluster**	**A1**	**A2**	**Self-fertile**	**Sample size**	**Χ**^**2**^ **-test**
LH	0.00	0.00	1.00	45	425.64*
QK	0.00	0.00	1.00	75	
XPII	0.00	0.00	1.00	127	
LY	0.49	0.00	0.51	101	
FZ	0.00	0.00	1.00	38	
ZZ	1.00	0.00	0.00	30	
CL	1.00	0.00	0.00	22	
XPI	1.00	0.00	0.00	96	
Cluster A	0.00	0.00	1.00	247	353.30*
Cluster B	0.47	0.00	0.53	169	
Cluster C	1.00	0.00	0.00	118	
Total	0.39	0.00	0.61	534	

^*^Significance at *p *< 0.001.

**Table 4:** Pairwise comparison of population differentiation (*F*_ST_) among the eight populations of *P. infestans* from Fujian, China.

**Population**	**LH**	**ZZ**	**XPII**	**XPI**	**LY**	**QK**	**CL**
ZZ	0.255**						
XPII	0.020*	0.247**					
XPI	0.288**	0.228**	0.289**				
LY	0.253**	0.025*	0.260**	0.269**			
QK	0.027*	0.248**	0.006	0.280**	0.263**		
CL	0.310**	0.246**	0.307**	0.001	0.288**	0.301**	
FZ	0.222**	0.098**	0.229**	0.198**	0.095**	0.221**	0.212**

^*^Significantly larger than zero at *p *= 0.05.

^**^Significantly larger than zero at *p *= 0.01.

**Table 5:** Analysis of molecular variance (AMOVA) for the eight field populations and three clusters in the *P. infestans* from Fujian, China.

	**d.f.**	**SS**	**Variance**	**%**	***P*****-values**
Field populations					
Among years	2	347.37	0.47	21.42	0.013
Among populations within year	5	67.38	0.11	4.45	<0.001
Within population	1060	1756.96	1.66	74.12	<0.001
Total	1067	2171.61	2.24		

Clusters					
Among clusters	2	382.35	0.54	23.98	0.007
Among populations within cluster	5	32.3	0.04	1.91	<0.001
Within population	1060	1756.96	1.66	74.12	<0.001
Total	1067	2171.61	2.24		

## References

[b1] Calo S., Billmyre R. B. & Heitman J. Generators of phenotypic diversity in the evolution of pathogenic microorganisms. PLoS Pathog 9, e1003181 (2013). 23555239 10.1371/journal.ppat.1003181 PMC3605297

[b2] Bernstein H., Hopf F. A. & Michod R. E. The molecular basis of the evolution of sex. Adv. Genet. 24, 323–370 (1987). 3324702 10.1016/s0065-2660(08)60012-7

[b3] Nunney L. & Luck R. F. Factors influencing the optimum sex ratio in a structured population. Theor. Popul. Biol. 33, 1–30 (1988). 3376051 10.1016/0040-5809(88)90002-0

[b4] Ebert D. *et al.* A selective advantage to immigrant genes in a Daphnia metapopulation. Science 295, 485–488 (2002). 11799241 10.1126/science.1067485

[b5] Saccheri I. J. & Brakefield P. M. Rapid spread of immigrant genomes into inbred populations. Proc. R. Soc. Lond. B 269, 1073–1078 (2002). 10.1098/rspb.2002.1963 PMC1690988 12028766

[b6] Barton N. & Bengtsson B. O. The barrier to genetic exchange between hybridising populations. Heredity 57, 357–376 (1986). 3804765 10.1038/hdy.1986.135

[b7] Barrett L. G., Thrall P. H., Burdon J. J. & Linde C. C. Life history determines genetic structure and evolutionary potential of host-parasite interactions. Trends. Ecol. Evol 23, 678–685 (2008). 18947899 10.1016/j.tree.2008.06.017 PMC2653456

[b8] Zhan J., Pettway R. E. & McDonald B. A. The global genetic structure of the wheat pathogen *Mycosphaerella graminicola* is characterized by high nuclear diversity, low mitochondrial diversity, regular recombination, and gene flow. Fungal Genet. Biol. 38, 286–297 (2003). 12684018 10.1016/s1087-1845(02)00538-8

[b9] Zhan J., Thrall P. H. & Burdon J. J. Achieving sustainable plant disease management through evolutionary principles. Trends. Plant Sci. 19, 570–575 (2014). 24853471 10.1016/j.tplants.2014.04.010

[b10] Bourke P. M. A. Emergence of potato blight. 1843-46. Nature 203, 805–808 (1964).

[b11] Fry W. *Phytophthora infestans*: the plant (and R gene) destroyer. Mol. Plant Pathol 9, 385–402 (2008). 18705878 10.1111/j.1364-3703.2007.00465.x PMC6640234

[b12] Haverkort A. J. *et al.* Societal costs of late blight in potato and prospects of durable resistance through cisgenic modification. Potato Res. 51, 47–57 (2008).

[b13] Wang Q. B. & Zhang W. China’s potato industry and potential impacts on the global market. Am. J. Potato Res. 81, 101–109 (2004).

[b14] Jansky S. H., Jin L. P., Xie K. Y., Xie C. H. & Spooner D. M. Potato production and breeding in China. Potato Res. 52, 57–65 (2009).

[b15] Wang B. *et al.* Potato viruses in China. Crop Pro. 30, 1117–1123 (2011).

[b16] Li Y. *et al.* Population structure of *Phytophthora infestans* in China - geographic clusters and presence of the EU genotype Blue-13. Plant Pathol. 62, 932–942 (2013).

[b17] Achbani E. H. *et al.* Potato late blight in Morocco: characterization of *Phytophthora infestans* populations (virulence and mating type). Commun. Agric. Appl. Biol. Sci. 70, 247–252 (2005). 16637185

[b18] Groves C. L. Characterization of *Phytophthora infestans* from Maine during 1999 and 2000. Am. J. Potato Res. 79, 325–333 (2002).

[b19] Han M. *et al.* *Phytophthora infestans* field isolates from Gansu province, China are genetically highly diverse and show a high frequency of self fertility. J. Eukaryot. Microbiol 60, 79–88 (2013). 23194320 10.1111/jeu.12010

[b20] Alarcon-Rodriguez N. M., Lozoya-Saldana H., Valadez-Moctezuma E., Garcia-Mateos M. D. & Colinas-Leon M. T. Genetic diversity of potato late blight [*Phytophthora infestans* (Mont) de Bary] at Chapingo, Mexico. Agrociencia 47, 593–607 (2013).

[b21] Orona C. A. L. *et al.* First Report of homothallic isolates of *Phytophthora infestans* in commercial potato crops (*Solanum tuberosum*) in the Toluca valley, Mexico. Plant Dis. 97, 1112–1112 (2013). 10.1094/PDIS-10-12-0962-PDN 30722511

[b22] Smoot J. J. *et al.* Production and germination of oospores of *Phytophthora infestan*s. Phytopathology 48, 165–171 (1958).

[b23] Yoshida K. *et al.* The rise and fall of the *Phytophthora infestans* lineage that triggered the Irish potato famine. eLife 2, e00731 (2013). 23741619 10.7554/eLife.00731 PMC3667578

[b24] Goodwin S. B. & Drenth A. Origin of the A2 mating type of *Phytophthora infestans* outside Mexico. Phytopathology 87, 992–999 (1997). 18945031 10.1094/PHYTO.1997.87.10.992

[b25] Cooke L. R. *et al.* The Northern Ireland *Phytophthora infestans* population 1998-2002 characterized by genotypic and phenotypic markers. Plant Pathol. 55, 320–330 (2006).

[b26] Montarry J. *et al.* Microsatellite markers reveal two admixed genetic groups and an ongoing displacement within the French population of the invasive plant pathogen *Phytophthora infestans*. Mol. Ecol. 19, 1965–1977 (2010). 20345671 10.1111/j.1365-294X.2010.04619.x

[b27] Pule B. B. *et al.* *Phytophthora infestans* populations in central, eastern and southern African countries consist of two major clonal lineages. Plant Pathol. 62, 154–165 (2013).

[b28] Seidl A. C. & Gevens A. J. Characterization and distribution of three new clonal lineages of *Phytophthora infestans* causing late blight in Wisconsin from 2009 to 2012. Am. J. Potato Res. 90, 551–560 (2013).

[b29] Brurberg M. B. *et al.* Genetic analysis of *Phytophthora infestans* populations in the Nordic European countries reveals high genetic variability. Fungal Biol. 115, 335–342 (2011). 21530915 10.1016/j.funbio.2011.01.003

[b30] McDonald B. A. & Linde C. Pathogen population genetics, evolutionary potential, and durable resistance. Annu. Rev. Phytopathol 40, 349–379 (2002). 12147764 10.1146/annurev.phyto.40.120501.101443

[b31] Day J. P. & Shattock R. C. Aggressiveness and other factors relating to displacement of populations of *Phytophthora infestans* in England and Wales. Eur. J. Plant Pathol. 103, 379–391 (1997).

[b32] Kato M., Mizubuti E. S., Goodwin S. B. & Fry W. E. Sensitivity to protectant fungicides and pathogenic fitness of clonal lineages of *Phytophthora infestans* in the United States. Phytopathology 87, 973–978 (1997). 18945070 10.1094/PHYTO.1997.87.9.973

[b33] Cooke D. E. L. *et al.* Genome analyses of an aggressive and invasive lineage of the Irish potato famine pathogen. PloS Pathog. 8, e1002940 (2012). 23055926 10.1371/journal.ppat.1002940 PMC3464212

[b34] Li Y. *et al.* Population dynamics of *Phytophthora infestans* in the Netherlands reveals expansion and spread of dominant clonal lineages and virulence in sexual offspring. G3-Genes Genom. Genet. 2, 1529–1540 (2012). 10.1534/g3.112.004150 PMC3516475 23275876

[b35] Gisi U., Walder F., Resheat-Eini Z., Edel D. & Sierotzki H. Changes of genotype, sensitivity and aggressiveness in *Phytophthora infestans* isolates collected in European countries in 1997, 2006 and 2007. J. Phytopathol. 159, 223–232 (2011).

[b36] Goss E. M. *et al.* The Irish potato famine pathogen *Phytophthora infestans* originated in central Mexico rather than the Andes. Proc. Natl. Acad. Sci. USA 111, 8791–8796 (2014). 24889615 10.1073/pnas.1401884111 PMC4066499

[b37] Li B. *et al.* Phenotypic and genotypic characterization of *Phytophthora infestans* isolates from China. J. Phytopathol. 157, 558–567 (2009).

[b38] Zhan J., Torriani S. F. F. & McDonald B. A. Significant difference in pathogenicity between *MAT1-1* and *MAT1-2* isolates in the wheat pathogen *Mycosphaerella graminicola*. Fungal Genet. Biol. 44, 339–346 (2007). 17157539 10.1016/j.fgb.2006.10.008

[b39] Smart C. D., Mayton H., Mizubuti E. S., Willmann M. R. & Fry W. E. Environmental and genetic factors influencing self-fertility in *Phytophthora infestans*. Phytopathology 90, 987–994 (2000). 18944524 10.1094/PHYTO.2000.90.9.987

[b40] Chmielarz M. *et al.* Diversity of *Phytophthora infestans* from Poland. Plant Pathol. 63, 203–211 (2014).

[b41] Zwankhuizen M. J., Govers F. & Zadoks J. C. Development of potato late blight epidemics: disease foci, disease gradients, and infection sources. Phytopathology 88, 754–763 (1998). 18944880 10.1094/PHYTO.1998.88.8.754

[b42] Kimura M. & Weiss G. H. The stepping stone model of population structure and the decrease of genetic correlation with distance. Genetics 49, 561–576 (1964). 17248204 10.1093/genetics/49.4.561 PMC1210594

[b43] Irwin A. J. & Taylor P. D. Evolution of dispersal in a stepping-stone population with overlapping generations. Theor. Popul. Biol. 58, 321–328 (2000). 11162790 10.1006/tpbi.2000.1490

[b44] Slatkin M. Gene flow and the geographic structure of natural populations. Science 236, 787–792 (1987). 3576198 10.1126/science.3576198

[b45] Muller H. J. The relation of recombination to mutational advance. Mutat. Res. 106, 2–9 (1964). 14195748 10.1016/0027-5107(64)90047-8

[b46] Caten C. E. & Jinks J. L. Spontaneous variability of single isolates of *Phytophthora infestans*. I. Cultural variation. Can. J. Bot. 46, 329–348 (1968).

[b47] Knapova G. & Gisi U. Phenotypic and genotypic structure of *Phytophthora infestans* populations on potato and tomato in France and Switzerland. Plant Pathol. 51, 641–653 (2002).

[b48] Lees A. K. *et al.* Novel microsatellite markers for the analysis of *Phytophthora infestans* populations. Plant Pathol. 55, 311–319 (2006).

[b49] Arnaud-Haond S. & Belkhir K. GENCLONE: a computer program to analyse genotypic data, test for clonality and describe spatial clonal organization. Mol. Ecol. Notes. 7, 15–17 (2007).

[b50] Arnaud-Haond S., Duarte C. M., Alberto F. & Serrao E. A. Standardizing methods to address clonality in population studies. Mol. Ecol. 16, 5115–5139 (2007). 17944846 10.1111/j.1365-294X.2007.03535.x

[b51] Salipante S.J. & Hall B.G. Inadequacies of minimum spanning trees in molecular epidemiology. J. Clin. Microbiol. 49, 3568–3575 (2011). 21849692 10.1128/JCM.00919-11 PMC3187300

