# Supplementary material for: Limited Sexual Reproduction and Quick Turnover in the Population Genetic Structure of Phytophthora infestans in Fujian, China
Source: Sci Rep. 2015 May 13;5:10094. doi: 10.1038/srep10094 (PMC4429539; doi:10.1038/srep10094)
Supplement: Supplementary Information [file srep10094-s1.pdf]

# Limited Sexual Reproduction and Quick Turnover in the Population Genetic Structure of *Phytophthora infestans* in Fujian, China

Wen Zhu[1#], Li-Na Yang[1#], E-Jiao Wu[1], Chun-Fang Qin[1], Li-Ping Shang[1], Zong-Hua Wang[2] and Jiasui Zhan[2*]

[1]Fujian Key Lab of Plant Virology, Institute of Plant Virology, Fujian Agriculture and Forestry University, Fuzhou, Fujian, P. R. China

[2]Key Lab for Biopesticide and Chemical Biology, Ministry of Education, Fujian Agriculture and Forestry University, Fuzhou, Fujian, P. R. China

* Corresponding author

Supplementary table 1. SSR of *Phytophthora infestans* isolates from Fujian province in China tested in this study.

| POP | Sample | G11 | G11 | PI56 | PI56 | PI33 | PI33 | PI04 | PI04 | PI89 | PI89 | PI4B | PI4B | PI02 | PI02 | PI16 | PI16 |
|---|---|---|---|---|---|---|---|---|---|---|---|---|---|---|---|---|---|
| LH | LH10 | 160 | 162 | 173 | 175 | 203 | 203 | 168 | 172 | 177 | 177 | 207 | 215 | 162 | 164 | 174 | 176 |
| LH | LH11 | 160 | 162 | 173 | 175 | 203 | 203 | 168 | 172 | 175 | 177 | 207 | 215 | 162 | 164 | 174 | 176 |
| LH | LH12 | 160 | 162 | 173 | 175 | 203 | 203 | 168 | 172 | 177 | 177 | 207 | 215 | 162 | 164 | 176 | 176 |
| LH | LH13 | 160 | 162 | 173 | 175 | 203 | 203 | 168 | 172 | 177 | 177 | 207 | 215 | 162 | 164 | 176 | 176 |
| LH | LH14 | 160 | 162 | 173 | 175 | 203 | 203 | 168 | 172 | 177 | 177 | 207 | 215 | 162 | 164 | 174 | 176 |
| LH | LH15 | 160 | 162 | 173 | 175 | 203 | 203 | 168 | 172 | 177 | 177 | 207 | 215 | 162 | 164 | 174 | 176 |
| LH | LH16 | 160 | 162 | 173 | 175 | 203 | 203 | 168 | 172 | 177 | 177 | 207 | 215 | 162 | 164 | 174 | 176 |
| LH | LH18 | 160 | 162 | 173 | 175 | 203 | 203 | 168 | 172 | 177 | 177 | 207 | 215 | 162 | 164 | 174 | 176 |
| LH | LH19 | 160 | 162 | 173 | 175 | 203 | 203 | 168 | 172 | 177 | 177 | 207 | 215 | 162 | 164 | 174 | 176 |
| LH | LH2 | 160 | 162 | 173 | 175 | 203 | 203 | 168 | 172 | 177 | 177 | 207 | 215 | 162 | 164 | 176 | 176 |
| LH | LH20 | 160 | 162 | 173 | 175 | 203 | 203 | 168 | 172 | 177 | 177 | 207 | 215 | 162 | 164 | 174 | 176 |
| LH | LH21 | 160 | 162 | 173 | 175 | 203 | 203 | 168 | 172 | 177 | 177 | 207 | 215 | 162 | 164 | 176 | 176 |
| LH | LH22 | 160 | 162 | 173 | 175 | 203 | 203 | 168 | 172 | 177 | 177 | 207 | 215 | 162 | 164 | 176 | 176 |
| LH | LH23 | 160 | 162 | 173 | 175 | 203 | 203 | 168 | 172 | 177 | 177 | 207 | 215 | 162 | 164 | 176 | 176 |
| LH | LH24 | 160 | 162 | 173 | 175 | 203 | 203 | 168 | 172 | 177 | 177 | 207 | 215 | 162 | 164 | 176 | 176 |
| LH | LH25 | 160 | 162 | 173 | 175 | 203 | 203 | 168 | 172 | 177 | 177 | 207 | 215 | 162 | 164 | 174 | 176 |
| LH | LH26 | 160 | 162 | 173 | 175 | 203 | 203 | 168 | 172 | 177 | 177 | 207 | 215 | 162 | 164 | 176 | 176 |
| LH | LH27 | 160 | 162 | 173 | 175 | 203 | 203 | 168 | 172 | 177 | 177 | 207 | 215 | 162 | 164 | 176 | 176 |
| LH | LH28 | 160 | 162 | 173 | 175 | 203 | 203 | 168 | 172 | 177 | 177 | 207 | 215 | 162 | 164 | 176 | 176 |
| LH | LH29 | 160 | 162 | 173 | 175 | 203 | 203 | 168 | 172 | 177 | 177 | 207 | 215 | 162 | 164 | 174 | 176 |
| LH | LH3 | 160 | 162 | 173 | 175 | 203 | 203 | 168 | 172 | 177 | 177 | 207 | 215 | 162 | 164 | 176 | 176 |
| LH | LH30 | 160 | 162 | 173 | 175 | 203 | 203 | 168 | 172 | 177 | 177 | 207 | 215 | 162 | 164 | 176 | 176 |
| LH | LH31 | 160 | 162 | 173 | 175 | 203 | 203 | 168 | 172 | 177 | 177 | 207 | 215 | 162 | 164 | 174 | 176 |
| LH | LH32 | 160 | 162 | 173 | 175 | 203 | 203 | 168 | 172 | 177 | 177 | 207 | 215 | 162 | 164 | 176 | 176 |
| LH | LH33 | 160 | 162 | 173 | 175 | 203 | 203 | 168 | 172 | 177 | 177 | 207 | 215 | 162 | 164 | 176 | 176 |
| LH | LH34 | 160 | 162 | 173 | 175 | 203 | 203 | 168 | 172 | 177 | 179 | 207 | 215 | 162 | 164 | 176 | 176 |
| LH | LH35 | 160 | 162 | 173 | 175 | 203 | 203 | 168 | 172 | 177 | 177 | 207 | 215 | 162 | 164 | 174 | 176 |
| LH | LH36 | 156 | 158 | 173 | 175 | 203 | 203 | 168 | 172 | 177 | 179 | 207 | 219 | 162 | 164 | 176 | 176 |

| | | | | | | | | | | | | | | | | | |
|---|---|---|---|---|---|---|---|---|---|---|---|---|---|---|---|---|---|
| LH | LH37 | 160 | 162 | 173 | 175 | 203 | 203 | 168 | 172 | 177 | 177 | 207 | 215 | 162 | 164 | 174 | 176 |
| LH | LH38 | 160 | 162 | 173 | 175 | 203 | 203 | 168 | 172 | 177 | 179 | 207 | 215 | 162 | 164 | 174 | 176 |
| LH | LH39 | 160 | 162 | 173 | 175 | 203 | 203 | 168 | 172 | 177 | 177 | 207 | 215 | 162 | 164 | 176 | 176 |
| LH | LH4 | 160 | 162 | 173 | 175 | 203 | 203 | 168 | 172 | 177 | 177 | 207 | 215 | 162 | 164 | 174 | 176 |
| LH | LH40 | 160 | 162 | 173 | 175 | 203 | 203 | 168 | 172 | 177 | 177 | 207 | 215 | 162 | 164 | 176 | 176 |
| LH | LH41 | 160 | 162 | 173 | 175 | 203 | 203 | 168 | 172 | 177 | 177 | 207 | 215 | 162 | 164 | 176 | 176 |
| LH | LH42 | 160 | 162 | 173 | 175 | 203 | 203 | 168 | 172 | 177 | 177 | 207 | 215 | 162 | 164 | 176 | 176 |
| LH | LH43 | 160 | 162 | 173 | 175 | 203 | 203 | 168 | 172 | 177 | 177 | 207 | 215 | 162 | 164 | 176 | 176 |
| LH | LH44 | 160 | 162 | 173 | 175 | 203 | 203 | 168 | 172 | 177 | 177 | 207 | 215 | 162 | 164 | 174 | 176 |
| LH | LH45 | 160 | 162 | 173 | 175 | 203 | 203 | 168 | 172 | 177 | 177 | 207 | 215 | 162 | 164 | 174 | 176 |
| LH | LH46 | 160 | 162 | 173 | 175 | 203 | 203 | 168 | 172 | 177 | 177 | 207 | 215 | 162 | 164 | 176 | 176 |
| LH | LH47 | 160 | 162 | 173 | 175 | 203 | 203 | 168 | 172 | 177 | 177 | 207 | 215 | 162 | 164 | 176 | 176 |
| LH | LH48 | 160 | 162 | 173 | 175 | 203 | 203 | 168 | 172 | 177 | 177 | 207 | 215 | 162 | 164 | 176 | 176 |
| LH | LH49 | 160 | 162 | 173 | 175 | 203 | 203 | 168 | 172 | 177 | 177 | 207 | 215 | 162 | 164 | 176 | 176 |
| LH | LH6 | 160 | 162 | 173 | 175 | 203 | 203 | 168 | 172 | 177 | 177 | 207 | 215 | 162 | 164 | 176 | 176 |
| LH | LH9 | 160 | 162 | 173 | 175 | 203 | 203 | 168 | 172 | 177 | 177 | 207 | 215 | 162 | 164 | 174 | 176 |
| LH | LHB3-1 | 160 | 162 | 173 | 175 | 203 | 203 | 168 | 172 | 177 | 177 | 207 | 215 | 162 | 164 | 174 | 176 |
| ZZ | F38 | 156 | 158 | 175 | 175 | 203 | 206 | 168 | 172 | 177 | 179 | 207 | 219 | 162 | 164 | 174 | 176 |
| ZZ | F39 | 156 | 158 | 173 | 173 | 203 | 203 | 168 | 172 | 177 | 179 | 215 | 215 | 154 | 162 | 176 | 176 |
| ZZ | F40 | 156 | 158 | 175 | 175 | 203 | 206 | 168 | 172 | 177 | 179 | 207 | 219 | 162 | 164 | 174 | 176 |
| ZZ | F41 | 156 | 158 | 173 | 175 | 203 | 203 | 168 | 172 | 177 | 179 | 215 | 215 | 154 | 162 | 176 | 176 |
| ZZ | F42 | 156 | 158 | 173 | 175 | 203 | 203 | 168 | 172 | 177 | 179 | 215 | 215 | 154 | 162 | 176 | 176 |
| ZZ | F43 | 158 | 158 | 175 | 175 | 203 | 206 | 168 | 172 | 177 | 177 | 215 | 215 | 162 | 164 | 174 | 176 |
| ZZ | F44 | 156 | 158 | 173 | 175 | 203 | 203 | 168 | 172 | 177 | 179 | 215 | 215 | 154 | 162 | 176 | 176 |
| ZZ | F45 | 156 | 158 | 173 | 175 | 203 | 203 | 168 | 172 | 177 | 179 | 215 | 215 | 154 | 162 | 174 | 176 |
| ZZ | F46 | 156 | 158 | 173 | 175 | 203 | 203 | 168 | 172 | 177 | 179 | 215 | 215 | 154 | 162 | 174 | 176 |
| ZZ | F47 | 156 | 158 | 173 | 175 | 203 | 203 | 168 | 172 | 177 | 179 | 215 | 215 | 154 | 162 | 174 | 176 |
| ZZ | F48 | 156 | 158 | 173 | 175 | 203 | 203 | 168 | 172 | 177 | 177 | 215 | 215 | 154 | 162 | 174 | 176 |
| ZZ | F49 | 156 | 158 | 173 | 175 | 203 | 203 | 168 | 172 | 177 | 179 | 215 | 215 | 154 | 162 | 174 | 176 |
| ZZ | F50 | 156 | 158 | 173 | 175 | 203 | 203 | 168 | 172 | 177 | 179 | 215 | 215 | 154 | 162 | 174 | 176 |
| ZZ | F51 | 156 | 158 | 173 | 175 | 203 | 203 | 168 | 172 | 177 | 177 | 215 | 215 | 154 | 162 | 174 | 176 |

| | | | | | | | | | | | | | | | | | |
|---|---|---|---|---|---|---|---|---|---|---|---|---|---|---|---|---|---|
| ZZ | F52 | 156 | 158 | 173 | 175 | 203 | 203 | 168 | 172 | 177 | 179 | 215 | 215 | 154 | 162 | 176 | 176 |
| ZZ | F53 | 156 | 158 | 173 | 175 | 203 | 203 | 168 | 172 | 177 | 179 | 215 | 215 | 154 | 162 | 174 | 176 |
| ZZ | F54 | 156 | 158 | 173 | 175 | 203 | 203 | 168 | 172 | 177 | 179 | 215 | 215 | 154 | 160 | 176 | 176 |
| ZZ | F55 | 156 | 158 | 173 | 175 | 203 | 203 | 168 | 172 | 177 | 179 | 215 | 215 | 154 | 162 | 174 | 176 |
| ZZ | F56 | 156 | 158 | 173 | 175 | 203 | 203 | 168 | 172 | 177 | 179 | 215 | 215 | 154 | 162 | 174 | 176 |
| ZZ | F57 | 156 | 158 | 175 | 175 | 203 | 203 | 168 | 172 | 177 | 179 | 215 | 215 | 154 | 162 | 174 | 176 |
| ZZ | F58 | 156 | 156 | 173 | 175 | 203 | 203 | 168 | 172 | 177 | 179 | 215 | 215 | 154 | 162 | 174 | 176 |
| ZZ | F59 | 156 | 158 | 173 | 175 | 203 | 203 | 168 | 172 | 177 | 179 | 215 | 215 | 154 | 162 | 174 | 176 |
| ZZ | F60 | 156 | 158 | 175 | 175 | 203 | 206 | 168 | 172 | 177 | 179 | 207 | 219 | 162 | 162 | 174 | 176 |
| ZZ | F61 | 156 | 158 | 173 | 175 | 203 | 203 | 168 | 172 | 177 | 179 | 215 | 215 | 154 | 162 | 174 | 176 |
| ZZ | F62 | 156 | 158 | 173 | 175 | 203 | 203 | 168 | 172 | 177 | 179 | 215 | 215 | 154 | 162 | 174 | 176 |
| ZZ | F63 | 156 | 158 | 173 | 175 | 203 | 203 | 168 | 172 | 177 | 179 | 215 | 215 | 154 | 162 | 174 | 176 |
| ZZ | F64 | 156 | 158 | 173 | 175 | 203 | 203 | 168 | 172 | 177 | 179 | 215 | 215 | 154 | 162 | 174 | 176 |
| ZZ | F65 | 156 | 158 | 173 | 175 | 203 | 203 | 168 | 172 | 177 | 179 | 215 | 215 | 154 | 162 | 174 | 176 |
| ZZ | F66 | 156 | 158 | 173 | 175 | 203 | 203 | 168 | 172 | 177 | 179 | 215 | 215 | 154 | 162 | 174 | 176 |
| ZZ | F67 | 156 | 158 | 173 | 175 | 203 | 203 | 168 | 172 | 177 | 179 | 215 | 215 | 154 | 162 | 174 | 176 |
| XPⅡ | XPⅡ1 | 160 | 160 | 173 | 175 | 203 | 203 | 168 | 172 | 177 | 177 | 207 | 215 | 160 | 162 | 174 | 176 |
| XPⅡ | XPⅡ10 | 162 | 162 | 173 | 175 | 203 | 203 | 168 | 172 | 177 | 177 | 207 | 215 | 162 | 164 | 174 | 176 |
| XPⅡ | XPⅡ100 | 160 | 160 | 173 | 175 | 203 | 203 | 168 | 172 | 177 | 177 | 207 | 215 | 160 | 162 | 174 | 176 |
| XPⅡ | XPⅡ102 | 160 | 162 | 173 | 175 | 203 | 203 | 168 | 172 | 177 | 177 | 207 | 215 | 162 | 164 | 174 | 176 |
| XPⅡ | XPⅡ103 | 160 | 162 | 173 | 175 | 203 | 203 | 168 | 172 | 177 | 177 | 207 | 215 | 162 | 164 | 174 | 176 |
| XPⅡ | XPⅡ104 | 160 | 160 | 173 | 175 | 203 | 203 | 168 | 172 | 177 | 177 | 207 | 215 | 160 | 162 | 174 | 176 |
| XPⅡ | XPⅡ105 | 160 | 160 | 173 | 175 | 203 | 203 | 168 | 172 | 177 | 177 | 207 | 215 | 160 | 162 | 174 | 176 |
| XPⅡ | XPⅡ106 | 160 | 162 | 183 | 175 | 203 | 203 | 168 | 172 | 177 | 177 | 207 | 215 | 162 | 164 | 176 | 176 |
| XPⅡ | XPⅡ107 | 160 | 162 | 173 | 175 | 203 | 203 | 168 | 172 | 177 | 177 | 207 | 215 | 162 | 164 | 174 | 176 |
| XPⅡ | XPⅡ108 | 160 | 160 | 173 | 175 | 203 | 203 | 168 | 172 | 177 | 177 | 207 | 215 | 160 | 162 | 174 | 176 |
| XPⅡ | XPⅡ109 | 162 | 162 | 173 | 175 | 203 | 203 | 168 | 172 | 177 | 177 | 207 | 215 | 162 | 164 | 174 | 176 |
| XPⅡ | XPⅡ11 | 160 | 160 | 173 | 175 | 203 | 203 | 168 | 172 | 177 | 177 | 207 | 215 | 160 | 162 | 174 | 176 |
| XPⅡ | XPⅡ111 | 162 | 162 | 173 | 175 | 203 | 203 | 168 | 172 | 177 | 177 | 207 | 215 | 162 | 164 | 174 | 176 |
| XPⅡ | XPⅡ112 | 160 | 162 | 173 | 175 | 203 | 203 | 168 | 172 | 177 | 177 | 207 | 215 | 162 | 164 | 174 | 176 |
| XPⅡ | XPⅡ114 | 160 | 162 | 173 | 175 | 203 | 203 | 168 | 172 | 177 | 177 | 207 | 215 | 162 | 164 | 174 | 176 |

| | | | | | | | | | | | | | | | | | |
|---|---|---|---|---|---|---|---|---|---|---|---|---|---|---|---|---|---|
| XPⅡ | XPⅡ115 | 160 | 162 | 173 | 175 | 203 | 203 | 168 | 172 | 177 | 177 | 207 | 215 | 162 | 164 | 174 | 176 |
| XPⅡ | XPⅡ116 | 160 | 162 | 173 | 175 | 203 | 203 | 168 | 172 | 177 | 177 | 207 | 215 | 162 | 164 | 174 | 176 |
| XPⅡ | XPⅡ117 | 160 | 162 | 173 | 175 | 203 | 203 | 168 | 172 | 177 | 177 | 207 | 215 | 162 | 164 | 174 | 176 |
| XPⅡ | XPⅡ118 | 160 | 162 | 173 | 175 | 203 | 203 | 168 | 172 | 177 | 177 | 207 | 215 | 162 | 164 | 174 | 176 |
| XPⅡ | XPⅡ119 | 160 | 162 | 173 | 175 | 203 | 203 | 168 | 172 | 177 | 177 | 207 | 215 | 162 | 164 | 174 | 176 |
| XPⅡ | XPⅡ12 | 162 | 162 | 173 | 175 | 203 | 203 | 168 | 172 | 177 | 177 | 207 | 215 | 162 | 164 | 174 | 176 |
| XPⅡ | XPⅡ120 | 162 | 162 | 173 | 175 | 203 | 203 | 168 | 172 | 177 | 177 | 207 | 215 | 162 | 164 | 174 | 176 |
| XPⅡ | XPⅡ121 | 160 | 162 | 173 | 175 | 203 | 203 | 168 | 172 | 177 | 177 | 207 | 215 | 162 | 164 | 174 | 176 |
| XPⅡ | XPⅡ122 | 160 | 162 | 173 | 175 | 203 | 203 | 168 | 172 | 177 | 177 | 207 | 215 | 162 | 164 | 174 | 176 |
| XPⅡ | XPⅡ123 | 162 | 162 | 173 | 175 | 203 | 203 | 168 | 172 | 177 | 177 | 207 | 215 | 162 | 164 | 174 | 176 |
| XPⅡ | XPⅡ124 | 160 | 162 | 173 | 175 | 203 | 203 | 168 | 172 | 177 | 177 | 207 | 215 | 162 | 164 | 174 | 176 |
| XPⅡ | XPⅡ125 | 162 | 162 | 173 | 175 | 203 | 203 | 168 | 172 | 177 | 177 | 207 | 215 | 162 | 164 | 174 | 176 |
| XPⅡ | XPⅡ126 | 160 | 162 | 173 | 175 | 203 | 203 | 168 | 172 | 177 | 177 | 207 | 215 | 162 | 164 | 176 | 176 |
| XPⅡ | XPⅡ127 | 160 | 162 | 173 | 175 | 203 | 203 | 168 | 172 | 177 | 177 | 207 | 215 | 162 | 164 | 174 | 176 |
| XPⅡ | XPⅡ128 | 160 | 162 | 173 | 175 | 203 | 203 | 168 | 172 | 177 | 177 | 207 | 215 | 162 | 164 | 174 | 176 |
| XPⅡ | XPⅡ129 | 160 | 162 | 173 | 175 | 203 | 203 | 168 | 172 | 177 | 177 | 207 | 215 | 162 | 164 | 180 | 178 |
| XPⅡ | XPⅡ130 | 160 | 160 | 173 | 175 | 203 | 203 | 168 | 172 | 177 | 177 | 207 | 215 | 160 | 162 | 174 | 176 |
| XPⅡ | XPⅡ131 | 160 | 160 | 173 | 175 | 203 | 203 | 168 | 172 | 177 | 177 | 207 | 215 | 160 | 162 | 174 | 176 |
| XPⅡ | XPⅡ132 | 160 | 162 | 173 | 175 | 203 | 203 | 168 | 172 | 177 | 177 | 207 | 215 | 162 | 164 | 174 | 176 |
| XPⅡ | XPⅡ133 | 160 | 162 | 173 | 175 | 203 | 203 | 168 | 172 | 177 | 177 | 207 | 215 | 162 | 164 | 174 | 176 |
| XPⅡ | XPⅡ134 | 160 | 162 | 173 | 175 | 203 | 203 | 168 | 172 | 177 | 177 | 207 | 215 | 162 | 164 | 174 | 176 |
| XPⅡ | XPⅡ135 | 162 | 162 | 173 | 175 | 203 | 203 | 168 | 172 | 177 | 177 | 207 | 215 | 162 | 164 | 174 | 176 |
| XPⅡ | XPⅡ136 | 160 | 160 | 173 | 175 | 203 | 203 | 168 | 172 | 177 | 177 | 207 | 215 | 160 | 162 | 174 | 176 |
| XPⅡ | XPⅡ137 | 160 | 160 | 173 | 175 | 203 | 203 | 168 | 172 | 177 | 177 | 207 | 215 | 160 | 162 | 174 | 176 |
| XPⅡ | XPⅡ138 | 160 | 160 | 173 | 175 | 203 | 203 | 168 | 172 | 177 | 177 | 207 | 215 | 160 | 162 | 174 | 176 |
| XPⅡ | XPⅡ139 | 160 | 160 | 173 | 175 | 203 | 203 | 168 | 172 | 177 | 177 | 207 | 215 | 160 | 162 | 174 | 176 |
| XPⅡ | XPⅡ14 | 160 | 162 | 173 | 175 | 203 | 203 | 168 | 172 | 177 | 177 | 207 | 215 | 162 | 164 | 174 | 176 |
| XPⅡ | XPⅡ140 | 160 | 162 | 173 | 175 | 203 | 203 | 168 | 172 | 177 | 177 | 207 | 215 | 162 | 164 | 174 | 176 |
| XPⅡ | XPⅡ141 | 160 | 160 | 173 | 175 | 203 | 203 | 168 | 172 | 177 | 177 | 207 | 215 | 160 | 162 | 174 | 176 |
| XPⅡ | XPⅡ142 | 160 | 162 | 173 | 175 | 203 | 203 | 168 | 172 | 177 | 177 | 207 | 215 | 162 | 164 | 174 | 176 |
| XPⅡ | XPⅡ143 | 160 | 162 | 173 | 175 | 203 | 203 | 168 | 172 | 177 | 177 | 207 | 215 | 162 | 164 | 174 | 176 |

| | | | | | | | | | | | | | | | | | |
|---|---|---|---|---|---|---|---|---|---|---|---|---|---|---|---|---|---|
| XPⅡ | XPⅡ144 | 160 | 162 | 173 | 175 | 203 | 203 | 168 | 172 | 177 | 177 | 207 | 215 | 162 | 164 | 176 | 176 |
| XPⅡ | XPⅡ145 | 162 | 162 | 173 | 175 | 203 | 203 | 168 | 172 | 177 | 177 | 207 | 215 | 162 | 164 | 174 | 176 |
| XPⅡ | XPⅡ146 | 160 | 162 | 173 | 175 | 203 | 203 | 168 | 172 | 177 | 177 | 207 | 215 | 162 | 164 | 174 | 176 |
| XPⅡ | XPⅡ15 | 160 | 160 | 173 | 175 | 203 | 203 | 168 | 172 | 177 | 177 | 207 | 215 | 160 | 162 | 174 | 176 |
| XPⅡ | XPⅡ16 | 160 | 160 | 173 | 175 | 203 | 203 | 168 | 172 | 177 | 177 | 207 | 215 | 160 | 162 | 174 | 176 |
| XPⅡ | XPⅡ17 | 160 | 162 | 173 | 175 | 203 | 203 | 168 | 172 | 177 | 177 | 207 | 215 | 162 | 164 | 174 | 176 |
| XPⅡ | XPⅡ18 | 162 | 162 | 173 | 175 | 203 | 203 | 168 | 172 | 177 | 177 | 207 | 215 | 162 | 164 | 174 | 176 |
| XPⅡ | XPⅡ19 | 160 | 162 | 173 | 175 | 203 | 203 | 168 | 172 | 177 | 177 | 207 | 215 | 162 | 164 | 174 | 176 |
| XPⅡ | XPⅡ2 | 160 | 162 | 173 | 175 | 203 | 203 | 168 | 172 | 177 | 177 | 207 | 215 | 162 | 164 | 176 | 176 |
| XPⅡ | XPⅡ20 | 160 | 162 | 173 | 175 | 203 | 203 | 168 | 172 | 177 | 177 | 207 | 215 | 162 | 164 | 174 | 176 |
| XPⅡ | XPⅡ24 | 160 | 160 | 173 | 175 | 203 | 203 | 168 | 172 | 177 | 177 | 207 | 215 | 160 | 162 | 174 | 176 |
| XPⅡ | XPⅡ25 | 158 | 158 | 173 | 175 | 203 | 206 | 168 | 172 | 177 | 179 | 207 | 207 | 162 | 164 | 174 | 176 |
| XPⅡ | XPⅡ26 | 160 | 162 | 173 | 175 | 203 | 203 | 168 | 172 | 177 | 177 | 207 | 215 | 162 | 164 | 174 | 176 |
| XPⅡ | XPⅡ27 | 160 | 162 | 173 | 175 | 203 | 203 | 168 | 172 | 177 | 177 | 207 | 215 | 162 | 164 | 174 | 176 |
| XPⅡ | XPⅡ28 | 160 | 162 | 173 | 175 | 203 | 203 | 168 | 172 | 177 | 177 | 207 | 215 | 162 | 164 | 174 | 176 |
| XPⅡ | XPⅡ29 | 160 | 160 | 173 | 175 | 203 | 203 | 168 | 172 | 177 | 177 | 207 | 215 | 160 | 162 | 174 | 176 |
| XPⅡ | XPⅡ3 | 160 | 162 | 173 | 175 | 203 | 203 | 168 | 172 | 177 | 177 | 207 | 215 | 162 | 164 | 174 | 176 |
| XPⅡ | XPⅡ30 | 162 | 162 | 173 | 175 | 203 | 203 | 168 | 172 | 177 | 177 | 207 | 215 | 162 | 164 | 176 | 176 |
| XPⅡ | XPⅡ31 | 160 | 160 | 173 | 175 | 203 | 203 | 168 | 172 | 177 | 177 | 207 | 215 | 160 | 162 | 174 | 176 |
| XPⅡ | XPⅡ32 | 160 | 162 | 173 | 175 | 203 | 203 | 168 | 172 | 177 | 177 | 207 | 215 | 162 | 164 | 176 | 176 |
| XPⅡ | XPⅡ34 | 162 | 162 | 173 | 175 | 203 | 203 | 168 | 172 | 177 | 177 | 207 | 215 | 162 | 164 | 174 | 176 |
| XPⅡ | XPⅡ36 | 160 | 162 | 173 | 175 | 203 | 203 | 168 | 172 | 177 | 177 | 207 | 215 | 162 | 164 | 176 | 176 |
| XPⅡ | XPⅡ37 | 160 | 162 | 173 | 175 | 203 | 203 | 168 | 172 | 177 | 177 | 207 | 215 | 162 | 164 | 174 | 176 |
| XPⅡ | XPⅡ38 | 160 | 162 | 173 | 175 | 203 | 203 | 168 | 172 | 177 | 177 | 207 | 215 | 162 | 164 | 174 | 176 |
| XPⅡ | XPⅡ39 | 162 | 162 | 173 | 175 | 203 | 203 | 168 | 172 | 177 | 177 | 207 | 215 | 162 | 164 | 174 | 176 |
| XPⅡ | XPⅡ4 | 162 | 162 | 173 | 175 | 203 | 203 | 168 | 172 | 177 | 177 | 207 | 215 | 162 | 164 | 174 | 176 |
| XPⅡ | XPⅡ40 | 160 | 162 | 173 | 175 | 203 | 203 | 168 | 172 | 177 | 177 | 207 | 215 | 162 | 164 | 174 | 176 |
| XPⅡ | XPⅡ41 | 160 | 162 | 173 | 175 | 203 | 203 | 168 | 172 | 177 | 177 | 207 | 215 | 162 | 164 | 174 | 176 |
| XPⅡ | XPⅡ42 | 160 | 160 | 173 | 175 | 203 | 203 | 168 | 172 | 177 | 177 | 207 | 215 | 160 | 162 | 174 | 176 |
| XPⅡ | XPⅡ43 | 160 | 162 | 173 | 175 | 203 | 203 | 168 | 172 | 177 | 177 | 207 | 215 | 162 | 164 | 176 | 176 |
| XPⅡ | XPⅡ44 | 162 | 162 | 173 | 175 | 203 | 203 | 168 | 172 | 177 | 177 | 207 | 215 | 162 | 164 | 174 | 176 |

| | | | | | | | | | | | | | | | | | |
|---|---|---|---|---|---|---|---|---|---|---|---|---|---|---|---|---|---|
| XPⅡ | XPⅡ45 | 160 | 162 | 173 | 175 | 203 | 203 | 168 | 172 | 177 | 177 | 207 | 215 | 162 | 164 | 174 | 176 |
| XPⅡ | XPⅡ46 | 160 | 162 | 173 | 175 | 203 | 203 | 168 | 172 | 177 | 177 | 207 | 215 | 162 | 164 | 174 | 176 |
| XPⅡ | XPⅡ48 | 160 | 162 | 173 | 175 | 203 | 203 | 168 | 172 | 177 | 177 | 207 | 207 | 162 | 164 | 174 | 176 |
| XPⅡ | XPⅡ5 | 160 | 162 | 173 | 175 | 203 | 203 | 168 | 172 | 177 | 177 | 207 | 215 | 162 | 164 | 174 | 176 |
| XPⅡ | XPⅡ50 | 160 | 162 | 173 | 175 | 203 | 203 | 168 | 172 | 177 | 177 | 207 | 215 | 162 | 164 | 174 | 176 |
| XPⅡ | XPⅡ51 | 162 | 162 | 175 | 175 | 203 | 203 | 168 | 172 | 177 | 177 | 207 | 215 | 162 | 164 | 174 | 176 |
| XPⅡ | XPⅡ53 | 160 | 162 | 173 | 175 | 203 | 203 | 168 | 172 | 177 | 177 | 207 | 215 | 162 | 164 | 174 | 176 |
| XPⅡ | XPⅡ54 | 160 | 162 | 175 | 175 | 203 | 203 | 168 | 172 | 177 | 177 | 207 | 215 | 162 | 164 | 174 | 176 |
| XPⅡ | XPⅡ56 | 162 | 162 | 173 | 175 | 203 | 203 | 168 | 172 | 177 | 177 | 207 | 215 | 162 | 164 | 174 | 176 |
| XPⅡ | XPⅡ57 | 160 | 162 | 173 | 175 | 203 | 203 | 168 | 172 | 177 | 177 | 207 | 215 | 162 | 164 | 174 | 176 |
| XPⅡ | XPⅡ58 | 160 | 162 | 173 | 175 | 203 | 203 | 168 | 172 | 177 | 177 | 207 | 215 | 162 | 164 | 174 | 176 |
| XPⅡ | XPⅡ59 | 160 | 162 | 173 | 175 | 203 | 203 | 168 | 172 | 177 | 177 | 207 | 215 | 162 | 164 | 174 | 176 |
| XPⅡ | XPⅡ6 | 160 | 162 | 173 | 175 | 203 | 203 | 168 | 172 | 177 | 177 | 207 | 215 | 162 | 164 | 174 | 176 |
| XPⅡ | XPⅡ60 | 160 | 162 | 173 | 175 | 203 | 203 | 168 | 172 | 177 | 177 | 207 | 215 | 162 | 164 | 174 | 176 |
| XPⅡ | XPⅡ61 | 160 | 162 | 175 | 175 | 203 | 203 | 168 | 172 | 177 | 177 | 207 | 215 | 162 | 164 | 174 | 176 |
| XPⅡ | XPⅡ63 | 160 | 162 | 173 | 175 | 203 | 203 | 168 | 172 | 177 | 177 | 207 | 215 | 162 | 164 | 174 | 176 |
| XPⅡ | XPⅡ64 | 160 | 162 | 173 | 175 | 203 | 203 | 168 | 172 | 177 | 177 | 207 | 215 | 162 | 164 | 174 | 176 |
| XPⅡ | XPⅡ67 | 160 | 162 | 173 | 175 | 203 | 203 | 168 | 172 | 177 | 177 | 207 | 215 | 162 | 164 | 176 | 176 |
| XPⅡ | XPⅡ68 | 162 | 162 | 173 | 175 | 203 | 203 | 168 | 172 | 177 | 177 | 207 | 215 | 162 | 164 | 174 | 176 |
| XPⅡ | XPⅡ69 | 160 | 160 | 173 | 175 | 203 | 203 | 168 | 172 | 177 | 177 | 207 | 215 | 160 | 162 | 174 | 176 |
| XPⅡ | XPⅡ7 | 160 | 160 | 173 | 175 | 203 | 203 | 168 | 172 | 177 | 177 | 207 | 215 | 160 | 162 | 174 | 176 |
| XPⅡ | XPⅡ71 | 160 | 162 | 173 | 175 | 203 | 203 | 168 | 172 | 177 | 177 | 207 | 215 | 162 | 164 | 174 | 176 |
| XPⅡ | XPⅡ72 | 160 | 162 | 173 | 175 | 203 | 203 | 168 | 172 | 177 | 177 | 207 | 215 | 162 | 164 | 174 | 176 |
| XPⅡ | XPⅡ73 | 160 | 162 | 173 | 175 | 203 | 203 | 168 | 172 | 177 | 177 | 207 | 215 | 162 | 164 | 176 | 176 |
| XPⅡ | XPⅡ74 | 160 | 160 | 173 | 175 | 203 | 203 | 168 | 172 | 177 | 177 | 207 | 215 | 160 | 162 | 174 | 176 |
| XPⅡ | XPⅡ75 | 162 | 162 | 173 | 175 | 203 | 203 | 168 | 172 | 177 | 177 | 207 | 215 | 162 | 164 | 174 | 176 |
| XPⅡ | XPⅡ76 | 160 | 160 | 173 | 175 | 203 | 203 | 168 | 172 | 177 | 177 | 207 | 215 | 160 | 162 | 174 | 176 |
| XPⅡ | XPⅡ77 | 162 | 162 | 173 | 175 | 203 | 203 | 168 | 172 | 177 | 177 | 207 | 215 | 162 | 164 | 174 | 176 |
| XPⅡ | XPⅡ78 | 160 | 162 | 173 | 175 | 203 | 203 | 168 | 172 | 177 | 177 | 207 | 215 | 162 | 164 | 176 | 176 |
| XPⅡ | XPⅡ79 | 162 | 162 | 173 | 175 | 203 | 203 | 168 | 172 | 177 | 177 | 207 | 215 | 162 | 164 | 174 | 176 |
| XPⅡ | XPⅡ8 | 160 | 160 | 173 | 175 | 203 | 203 | 168 | 172 | 177 | 177 | 207 | 215 | 160 | 162 | 174 | 176 |

| | | | | | | | | | | | | | | | | | |
|---|---|---|---|---|---|---|---|---|---|---|---|---|---|---|---|---|---|
| XPⅡ | XPⅡ80 | 160 | 162 | 173 | 175 | 203 | 203 | 168 | 172 | 177 | 177 | 207 | 215 | 160 | 162 | 174 | 176 |
| XPⅡ | XPⅡ81 | 160 | 162 | 173 | 175 | 203 | 203 | 168 | 172 | 177 | 177 | 207 | 215 | 162 | 164 | 174 | 176 |
| XPⅡ | XPⅡ82 | 162 | 162 | 173 | 175 | 203 | 203 | 168 | 172 | 177 | 177 | 207 | 215 | 162 | 164 | 174 | 176 |
| XPⅡ | XPⅡ83 | 160 | 162 | 173 | 175 | 203 | 203 | 168 | 172 | 177 | 177 | 207 | 215 | 162 | 164 | 176 | 176 |
| XPⅡ | XPⅡ84 | 160 | 162 | 173 | 175 | 203 | 203 | 168 | 172 | 177 | 177 | 207 | 215 | 162 | 164 | 174 | 176 |
| XPⅡ | XPⅡ85 | 160 | 162 | 173 | 175 | 203 | 203 | 168 | 172 | 177 | 177 | 207 | 215 | 162 | 164 | 174 | 176 |
| XPⅡ | XPⅡ86 | 162 | 162 | 173 | 175 | 203 | 203 | 168 | 172 | 177 | 177 | 207 | 215 | 162 | 164 | 174 | 176 |
| XPⅡ | XPⅡ87 | 160 | 162 | 173 | 175 | 203 | 203 | 168 | 172 | 177 | 177 | 207 | 215 | 162 | 164 | 174 | 176 |
| XPⅡ | XPⅡ88 | 160 | 160 | 173 | 175 | 203 | 203 | 168 | 172 | 177 | 177 | 207 | 215 | 160 | 162 | 174 | 176 |
| XPⅡ | XPⅡ9 | 160 | 162 | 173 | 175 | 203 | 203 | 168 | 172 | 177 | 177 | 207 | 215 | 162 | 164 | 174 | 176 |
| XPⅡ | XPⅡ91 | 162 | 162 | 173 | 175 | 203 | 203 | 168 | 172 | 177 | 177 | 207 | 215 | 162 | 164 | 174 | 176 |
| XPⅡ | XPⅡ92 | 162 | 162 | 173 | 175 | 203 | 203 | 168 | 172 | 177 | 177 | 207 | 215 | 162 | 164 | 174 | 176 |
| XPⅡ | XPⅡ93 | 160 | 162 | 173 | 175 | 203 | 203 | 168 | 172 | 177 | 177 | 207 | 215 | 162 | 164 | 174 | 176 |
| XPⅡ | XPⅡ94 | 160 | 162 | 173 | 175 | 203 | 203 | 168 | 172 | 177 | 177 | 207 | 215 | 162 | 164 | 176 | 176 |
| XPⅡ | XPⅡ95 | 160 | 162 | 173 | 175 | 203 | 203 | 168 | 172 | 177 | 177 | 207 | 215 | 162 | 164 | 174 | 176 |
| XPⅡ | XPⅡ96 | 160 | 160 | 173 | 175 | 203 | 203 | 168 | 172 | 177 | 177 | 207 | 215 | 160 | 162 | 174 | 176 |
| XPⅡ | XPⅡ97 | 160 | 162 | 173 | 175 | 203 | 203 | 168 | 172 | 177 | 177 | 207 | 215 | 162 | 164 | 174 | 176 |
| XPⅡ | XPⅡ98 | 160 | 162 | 173 | 175 | 203 | 203 | 168 | 172 | 177 | 177 | 207 | 215 | 162 | 164 | 176 | 176 |
| XPⅡ | XPⅡ99 | 162 | 162 | 173 | 175 | 203 | 203 | 168 | 172 | 177 | 177 | 207 | 215 | 162 | 164 | 174 | 176 |
| XPⅠ | XPⅠ101 | 156 | 158 | 175 | 175 | 203 | 203 | 168 | 172 | 177 | 179 | 207 | 219 | 162 | 164 | 174 | 176 |
| XPⅠ | XPⅠ102 | 156 | 158 | 173 | 175 | 203 | 206 | 168 | 172 | 177 | 179 | 207 | 219 | 162 | 164 | 174 | 176 |
| XPⅠ | XPⅠ107 | 156 | 158 | 175 | 175 | 203 | 206 | 168 | 172 | 177 | 179 | 207 | 219 | 162 | 164 | 174 | 176 |
| XPⅠ | XPⅠ108 | 156 | 158 | 175 | 175 | 203 | 206 | 168 | 172 | 177 | 179 | 207 | 219 | 162 | 164 | 174 | 176 |
| XPⅠ | XPⅠ109 | 156 | 158 | 175 | 175 | 203 | 206 | 168 | 172 | 177 | 179 | 207 | 219 | 162 | 164 | 174 | 176 |
| XPⅠ | XPⅠ110 | 156 | 158 | 175 | 175 | 203 | 206 | 168 | 172 | 177 | 179 | 207 | 219 | 162 | 164 | 174 | 176 |
| XPⅠ | XPⅠ111 | 156 | 158 | 175 | 175 | 203 | 206 | 168 | 172 | 177 | 179 | 207 | 219 | 162 | 164 | 174 | 176 |
| XPⅠ | XPⅠ112 | 156 | 158 | 175 | 175 | 203 | 206 | 168 | 172 | 177 | 179 | 207 | 219 | 162 | 164 | 174 | 176 |
| XPⅠ | XPⅠ114 | 156 | 158 | 175 | 175 | 203 | 206 | 168 | 172 | 177 | 179 | 207 | 219 | 162 | 164 | 174 | 176 |
| XPⅠ | XPⅠ116 | 156 | 158 | 175 | 175 | 203 | 206 | 168 | 172 | 177 | 179 | 207 | 219 | 162 | 164 | 174 | 176 |
| XPⅠ | XPⅠ117 | 156 | 158 | 175 | 175 | 203 | 206 | 168 | 172 | 177 | 179 | 207 | 219 | 162 | 164 | 174 | 176 |
| XPⅠ | XPⅠ118 | 156 | 158 | 175 | 175 | 203 | 206 | 168 | 172 | 177 | 179 | 207 | 219 | 162 | 164 | 174 | 176 |

| | | | | | | | | | | | | | | | | | |
|---|---|---|---|---|---|---|---|---|---|---|---|---|---|---|---|---|---|
| XP Ⅰ | XP Ⅰ 119 | 156 | 158 | 175 | 175 | 203 | 206 | 168 | 172 | 177 | 179 | 207 | 219 | 162 | 164 | 174 | 176 |
| XP Ⅰ | XP Ⅰ 12 | 156 | 158 | 175 | 175 | 203 | 206 | 168 | 172 | 177 | 179 | 207 | 219 | 162 | 164 | 174 | 176 |
| XP Ⅰ | XP Ⅰ 121 | 156 | 158 | 173 | 175 | 203 | 206 | 168 | 172 | 177 | 179 | 207 | 219 | 162 | 164 | 174 | 176 |
| XP Ⅰ | XP Ⅰ 122 | 156 | 158 | 175 | 175 | 203 | 206 | 168 | 172 | 177 | 179 | 207 | 219 | 162 | 164 | 174 | 176 |
| XP Ⅰ | XP Ⅰ 125 | 156 | 158 | 175 | 175 | 203 | 206 | 168 | 172 | 177 | 179 | 207 | 219 | 162 | 164 | 174 | 176 |
| XP Ⅰ | XP Ⅰ 127 | 156 | 158 | 175 | 175 | 203 | 206 | 168 | 172 | 177 | 179 | 207 | 219 | 162 | 164 | 174 | 176 |
| XP Ⅰ | XP Ⅰ 128 | 156 | 158 | 175 | 175 | 203 | 203 | 168 | 172 | 177 | 179 | 207 | 219 | 162 | 164 | 174 | 176 |
| XP Ⅰ | XP Ⅰ 129 | 156 | 158 | 175 | 175 | 203 | 206 | 168 | 172 | 177 | 179 | 207 | 219 | 162 | 164 | 174 | 176 |
| XP Ⅰ | XP Ⅰ 13 | 156 | 158 | 175 | 175 | 203 | 206 | 168 | 172 | 177 | 179 | 207 | 219 | 162 | 164 | 174 | 176 |
| XP Ⅰ | XP Ⅰ 130 | 156 | 158 | 175 | 175 | 203 | 206 | 168 | 172 | 177 | 179 | 207 | 219 | 162 | 164 | 174 | 176 |
| XP Ⅰ | XP Ⅰ 131 | 156 | 158 | 175 | 175 | 203 | 206 | 168 | 172 | 177 | 179 | 207 | 219 | 162 | 164 | 174 | 176 |
| XP Ⅰ | XP Ⅰ 134 | 156 | 158 | 175 | 175 | 203 | 206 | 168 | 172 | 177 | 179 | 207 | 219 | 162 | 164 | 174 | 176 |
| XP Ⅰ | XP Ⅰ 135 | 156 | 158 | 175 | 175 | 203 | 206 | 168 | 172 | 177 | 179 | 207 | 219 | 162 | 164 | 174 | 176 |
| XP Ⅰ | XP Ⅰ 136 | 156 | 158 | 173 | 175 | 203 | 206 | 168 | 172 | 177 | 179 | 207 | 219 | 162 | 164 | 174 | 176 |
| XP Ⅰ | XP Ⅰ 137 | 156 | 158 | 173 | 175 | 203 | 206 | 168 | 172 | 177 | 179 | 207 | 219 | 162 | 164 | 174 | 176 |
| XP Ⅰ | XP Ⅰ 139 | 156 | 158 | 175 | 175 | 203 | 206 | 168 | 172 | 177 | 179 | 207 | 219 | 162 | 164 | 174 | 176 |
| XP Ⅰ | XP Ⅰ 140 | 156 | 158 | 173 | 175 | 203 | 206 | 168 | 172 | 177 | 179 | 207 | 219 | 162 | 164 | 174 | 176 |
| XP Ⅰ | XP Ⅰ 141 | 156 | 158 | 173 | 175 | 203 | 206 | 168 | 172 | 177 | 179 | 207 | 219 | 162 | 164 | 174 | 176 |
| XP Ⅰ | XP Ⅰ 142 | 156 | 158 | 175 | 175 | 203 | 203 | 168 | 172 | 177 | 179 | 207 | 219 | 162 | 164 | 174 | 176 |
| XP Ⅰ | XP Ⅰ 143 | 156 | 158 | 175 | 175 | 203 | 206 | 168 | 172 | 177 | 179 | 207 | 219 | 162 | 164 | 174 | 176 |
| XP Ⅰ | XP Ⅰ 144 | 156 | 158 | 175 | 175 | 203 | 206 | 168 | 172 | 177 | 179 | 207 | 219 | 162 | 164 | 174 | 176 |
| XP Ⅰ | XP Ⅰ 145 | 156 | 158 | 175 | 175 | 203 | 206 | 168 | 172 | 177 | 179 | 207 | 219 | 162 | 164 | 174 | 176 |
| XP Ⅰ | XP Ⅰ 146 | 156 | 158 | 173 | 175 | 203 | 206 | 168 | 172 | 177 | 179 | 207 | 219 | 162 | 164 | 174 | 176 |
| XP Ⅰ | XP Ⅰ 15 | 156 | 158 | 175 | 175 | 203 | 206 | 168 | 172 | 177 | 179 | 207 | 219 | 162 | 164 | 174 | 176 |
| XP Ⅰ | XP Ⅰ 17 | 156 | 158 | 175 | 175 | 203 | 206 | 168 | 172 | 177 | 179 | 207 | 219 | 162 | 164 | 174 | 176 |
| XP Ⅰ | XP Ⅰ 18 | 156 | 158 | 175 | 175 | 203 | 206 | 168 | 172 | 177 | 179 | 207 | 219 | 162 | 164 | 174 | 176 |
| XP Ⅰ | XP Ⅰ 19 | 156 | 158 | 175 | 175 | 203 | 206 | 168 | 172 | 177 | 179 | 207 | 219 | 162 | 164 | 174 | 176 |
| XP Ⅰ | XP Ⅰ 20 | 156 | 158 | 175 | 175 | 203 | 206 | 168 | 172 | 177 | 179 | 207 | 219 | 162 | 164 | 174 | 176 |
| XP Ⅰ | XP Ⅰ 22 | 156 | 158 | 175 | 175 | 203 | 206 | 168 | 172 | 177 | 179 | 207 | 219 | 162 | 164 | 174 | 176 |
| XP Ⅰ | XP Ⅰ 24 | 156 | 158 | 175 | 175 | 203 | 206 | 168 | 172 | 177 | 179 | 207 | 219 | 162 | 164 | 174 | 176 |
| XP Ⅰ | XP Ⅰ 25 | 156 | 158 | 175 | 175 | 203 | 206 | 168 | 172 | 177 | 179 | 207 | 219 | 162 | 164 | 174 | 176 |

| | | | | | | | | | | | | | | | | | |
|---|---|---|---|---|---|---|---|---|---|---|---|---|---|---|---|---|---|
| XPⅠ | XPⅠ27 | 156 | 158 | 175 | 175 | 203 | 206 | 168 | 172 | 177 | 179 | 207 | 219 | 162 | 164 | 174 | 176 |
| XPⅠ | XPⅠ28 | 156 | 158 | 175 | 175 | 203 | 206 | 168 | 172 | 177 | 179 | 207 | 219 | 162 | 164 | 174 | 176 |
| XPⅠ | XPⅠ3 | 156 | 158 | 173 | 175 | 203 | 206 | 168 | 172 | 177 | 179 | 215 | 215 | 162 | 164 | 174 | 176 |
| XPⅠ | XPⅠ32 | 156 | 158 | 175 | 175 | 203 | 203 | 168 | 172 | 177 | 179 | 207 | 219 | 162 | 164 | 174 | 176 |
| XPⅠ | XPⅠ33 | 156 | 158 | 175 | 175 | 203 | 203 | 168 | 172 | 177 | 179 | 207 | 219 | 162 | 164 | 174 | 176 |
| XPⅠ | XPⅠ34 | 156 | 158 | 175 | 175 | 203 | 206 | 168 | 172 | 177 | 179 | 207 | 219 | 162 | 164 | 174 | 176 |
| XPⅠ | XPⅠ35 | 156 | 158 | 175 | 175 | 203 | 206 | 168 | 172 | 177 | 179 | 207 | 219 | 162 | 164 | 174 | 176 |
| XPⅠ | XPⅠ36 | 156 | 158 | 175 | 175 | 203 | 206 | 168 | 172 | 177 | 179 | 207 | 219 | 162 | 164 | 174 | 176 |
| XPⅠ | XPⅠ37 | 156 | 158 | 175 | 175 | 203 | 206 | 168 | 172 | 177 | 179 | 207 | 219 | 162 | 164 | 174 | 176 |
| XPⅠ | XPⅠ38 | 156 | 158 | 175 | 175 | 203 | 206 | 168 | 172 | 177 | 179 | 207 | 219 | 162 | 164 | 174 | 176 |
| XPⅠ | XPⅠ39 | 156 | 158 | 175 | 175 | 203 | 206 | 168 | 172 | 177 | 179 | 207 | 219 | 162 | 164 | 174 | 176 |
| XPⅠ | XPⅠ4 | 156 | 158 | 173 | 175 | 203 | 203 | 168 | 172 | 177 | 179 | 215 | 215 | 162 | 164 | 174 | 176 |
| XPⅠ | XPⅠ41 | 156 | 158 | 175 | 175 | 203 | 206 | 168 | 172 | 177 | 179 | 207 | 219 | 162 | 164 | 174 | 176 |
| XPⅠ | XPⅠ42 | 156 | 158 | 175 | 175 | 203 | 206 | 168 | 172 | 177 | 179 | 207 | 219 | 162 | 164 | 174 | 176 |
| XPⅠ | XPⅠ43 | 156 | 158 | 175 | 175 | 203 | 206 | 168 | 172 | 177 | 179 | 207 | 219 | 162 | 164 | 174 | 176 |
| XPⅠ | XPⅠ44 | 156 | 158 | 175 | 175 | 203 | 206 | 168 | 172 | 177 | 179 | 207 | 219 | 162 | 164 | 174 | 176 |
| XPⅠ | XPⅠ45 | 156 | 158 | 175 | 175 | 203 | 206 | 168 | 172 | 177 | 179 | 207 | 219 | 162 | 164 | 174 | 176 |
| XPⅠ | XPⅠ48 | 156 | 158 | 175 | 175 | 203 | 206 | 168 | 172 | 177 | 179 | 207 | 219 | 162 | 164 | 174 | 176 |
| XPⅠ | XPⅠ49 | 156 | 158 | 175 | 175 | 203 | 206 | 168 | 172 | 177 | 179 | 207 | 219 | 162 | 164 | 174 | 176 |
| XPⅠ | XPⅠ50 | 156 | 158 | 175 | 175 | 203 | 206 | 168 | 172 | 177 | 179 | 207 | 219 | 162 | 164 | 174 | 176 |
| XPⅠ | XPⅠ53 | 156 | 158 | 175 | 175 | 203 | 206 | 168 | 172 | 177 | 179 | 207 | 219 | 162 | 164 | 174 | 176 |
| XPⅠ | XPⅠ54 | 156 | 158 | 175 | 175 | 203 | 206 | 168 | 172 | 177 | 179 | 207 | 219 | 162 | 164 | 174 | 176 |
| XPⅠ | XPⅠ55 | 156 | 158 | 175 | 175 | 203 | 206 | 168 | 172 | 177 | 179 | 207 | 219 | 162 | 164 | 174 | 176 |
| XPⅠ | XPⅠ56 | 156 | 158 | 175 | 175 | 203 | 206 | 168 | 172 | 177 | 179 | 207 | 219 | 162 | 164 | 174 | 176 |
| XPⅠ | XPⅠ59 | 156 | 158 | 175 | 175 | 203 | 206 | 168 | 172 | 177 | 179 | 207 | 219 | 162 | 164 | 174 | 176 |
| XPⅠ | XPⅠ60 | 156 | 158 | 175 | 175 | 203 | 206 | 168 | 172 | 177 | 179 | 207 | 219 | 162 | 164 | 174 | 176 |
| XPⅠ | XPⅠ61 | 156 | 158 | 175 | 175 | 203 | 206 | 168 | 172 | 177 | 179 | 207 | 219 | 162 | 164 | 174 | 176 |
| XPⅠ | XPⅠ66 | 156 | 158 | 175 | 175 | 203 | 206 | 168 | 172 | 177 | 179 | 207 | 219 | 162 | 164 | 174 | 176 |
| XPⅠ | XPⅠ67 | 156 | 158 | 173 | 175 | 203 | 203 | 168 | 172 | 177 | 179 | 215 | 215 | 162 | 164 | 174 | 176 |
| XPⅠ | XPⅠ68 | 156 | 158 | 175 | 175 | 203 | 206 | 168 | 172 | 177 | 179 | 207 | 219 | 162 | 164 | 174 | 176 |
| XPⅠ | XPⅠ69 | 156 | 158 | 173 | 175 | 203 | 206 | 168 | 172 | 177 | 179 | 207 | 219 | 162 | 164 | 174 | 176 |

| | | | | | | | | | | | | | | | | | |
|---|---|---|---|---|---|---|---|---|---|---|---|---|---|---|---|---|---|
| XPⅠ | XPⅠ7 | 156 | 158 | 175 | 175 | 203 | 206 | 168 | 172 | 177 | 179 | 207 | 219 | 162 | 164 | 174 | 176 |
| XPⅠ | XPⅠ71 | 156 | 158 | 173 | 175 | 203 | 206 | 168 | 172 | 177 | 179 | 207 | 219 | 162 | 164 | 174 | 176 |
| XPⅠ | XPⅠ72 | 156 | 158 | 175 | 175 | 203 | 206 | 168 | 172 | 177 | 179 | 207 | 219 | 162 | 164 | 174 | 176 |
| XPⅠ | XPⅠ74 | 156 | 158 | 175 | 175 | 203 | 206 | 168 | 172 | 177 | 179 | 207 | 219 | 162 | 164 | 174 | 176 |
| XPⅠ | XPⅠ75 | 156 | 158 | 175 | 175 | 203 | 206 | 168 | 172 | 177 | 179 | 207 | 219 | 162 | 164 | 174 | 176 |
| XPⅠ | XPⅠ76 | 156 | 158 | 175 | 175 | 203 | 206 | 168 | 172 | 177 | 179 | 207 | 219 | 162 | 164 | 174 | 176 |
| XPⅠ | XPⅠ77 | 156 | 158 | 175 | 175 | 203 | 206 | 168 | 172 | 177 | 179 | 207 | 219 | 162 | 164 | 174 | 176 |
| XPⅠ | XPⅠ78 | 156 | 158 | 175 | 175 | 203 | 206 | 168 | 172 | 177 | 179 | 207 | 219 | 162 | 164 | 174 | 176 |
| XPⅠ | XPⅠ79 | 156 | 158 | 175 | 175 | 203 | 206 | 168 | 172 | 177 | 179 | 207 | 219 | 162 | 164 | 174 | 176 |
| XPⅠ | XPⅠ8 | 156 | 158 | 175 | 175 | 203 | 206 | 168 | 172 | 177 | 179 | 207 | 219 | 162 | 164 | 174 | 176 |
| XPⅠ | XPⅠ80 | 156 | 158 | 175 | 175 | 203 | 206 | 168 | 172 | 177 | 179 | 207 | 219 | 162 | 164 | 174 | 176 |
| XPⅠ | XPⅠ81 | 156 | 158 | 175 | 175 | 203 | 206 | 168 | 172 | 177 | 179 | 207 | 219 | 162 | 164 | 174 | 176 |
| XPⅠ | XPⅠ82 | 156 | 158 | 173 | 175 | 203 | 206 | 168 | 172 | 177 | 179 | 207 | 219 | 162 | 164 | 174 | 176 |
| XPⅠ | XPⅠ83 | 156 | 158 | 175 | 175 | 203 | 206 | 168 | 172 | 177 | 179 | 207 | 219 | 162 | 164 | 174 | 176 |
| XPⅠ | XPⅠ85 | 156 | 158 | 175 | 175 | 203 | 206 | 168 | 172 | 177 | 179 | 207 | 219 | 162 | 164 | 174 | 176 |
| XPⅠ | XPⅠ87 | 156 | 158 | 175 | 175 | 203 | 206 | 168 | 172 | 177 | 179 | 207 | 219 | 162 | 164 | 174 | 176 |
| XPⅠ | XPⅠ9 | 156 | 158 | 175 | 175 | 203 | 206 | 168 | 172 | 177 | 179 | 207 | 219 | 162 | 164 | 174 | 176 |
| XPⅠ | XPⅠ92 | 156 | 158 | 175 | 175 | 203 | 206 | 168 | 172 | 177 | 179 | 207 | 219 | 162 | 164 | 174 | 176 |
| XPⅠ | XPⅠ93 | 156 | 158 | 175 | 175 | 203 | 206 | 168 | 172 | 177 | 179 | 207 | 219 | 162 | 164 | 174 | 176 |
| XPⅠ | XPⅠ95 | 156 | 158 | 175 | 175 | 203 | 206 | 168 | 172 | 177 | 179 | 207 | 219 | 162 | 164 | 174 | 176 |
| XPⅠ | XPⅠ96 | 156 | 158 | 175 | 175 | 203 | 206 | 168 | 172 | 177 | 179 | 207 | 219 | 162 | 164 | 174 | 176 |
| XPⅠ | XPⅠ99 | 156 | 158 | 175 | 175 | 203 | 206 | 168 | 172 | 177 | 179 | 207 | 219 | 162 | 164 | 174 | 176 |
| LY | LY1 | 156 | 158 | 173 | 175 | 203 | 203 | 168 | 172 | 177 | 179 | 215 | 215 | 162 | 164 | 174 | 176 |
| LY | LY10 | 156 | 158 | 173 | 175 | 203 | 203 | 168 | 172 | 177 | 179 | 215 | 215 | 154 | 162 | 176 | 176 |
| LY | LY100 | 156 | 158 | 173 | 175 | 203 | 203 | 168 | 172 | 177 | 179 | 215 | 215 | 154 | 162 | 176 | 176 |
| LY | LY102 | 156 | 158 | 173 | 175 | 203 | 203 | 168 | 172 | 177 | 179 | 215 | 215 | 154 | 162 | 176 | 176 |
| LY | LY104 | 156 | 158 | 173 | 175 | 203 | 203 | 168 | 172 | 177 | 179 | 215 | 215 | 154 | 162 | 176 | 176 |
| LY | LY106 | 156 | 158 | 173 | 175 | 203 | 206 | 168 | 172 | 177 | 179 | 215 | 215 | 162 | 164 | 174 | 176 |
| LY | LY107 | 156 | 158 | 173 | 175 | 203 | 203 | 168 | 172 | 177 | 179 | 215 | 215 | 154 | 162 | 176 | 176 |
| LY | LY110 | 156 | 158 | 173 | 175 | 203 | 203 | 168 | 172 | 177 | 179 | 215 | 215 | 162 | 164 | 174 | 176 |
| LY | LY111 | 156 | 158 | 173 | 175 | 203 | 206 | 168 | 172 | 177 | 179 | 215 | 215 | 160 | 162 | 176 | 180 |

| | | | | | | | | | | | | | | | | | |
|---|---|---|---|---|---|---|---|---|---|---|---|---|---|---|---|---|---|
| LY | LY112 | 156 | 158 | 173 | 175 | 203 | 203 | 168 | 172 | 177 | 179 | 215 | 215 | 154 | 162 | 176 | 180 |
| LY | LY114 | 156 | 158 | 173 | 175 | 203 | 203 | 168 | 172 | 177 | 179 | 215 | 215 | 162 | 164 | 174 | 176 |
| LY | LY115 | 156 | 158 | 173 | 175 | 203 | 203 | 168 | 172 | 177 | 179 | 215 | 215 | 154 | 162 | 176 | 180 |
| LY | LY116 | 156 | 158 | 173 | 175 | 203 | 203 | 168 | 172 | 177 | 179 | 215 | 215 | 154 | 162 | 176 | 180 |
| LY | LY117 | 156 | 158 | 173 | 175 | 203 | 203 | 168 | 172 | 177 | 179 | 215 | 215 | 154 | 162 | 176 | 176 |
| LY | LY118 | 156 | 158 | 173 | 175 | 203 | 203 | 168 | 172 | 177 | 179 | 215 | 215 | 162 | 164 | 174 | 176 |
| LY | LY12 | 156 | 158 | 173 | 175 | 203 | 203 | 168 | 172 | 177 | 179 | 215 | 215 | 160 | 162 | 176 | 180 |
| LY | LY120 | 156 | 158 | 173 | 175 | 203 | 203 | 168 | 172 | 177 | 179 | 215 | 215 | 154 | 162 | 176 | 176 |
| LY | LY121 | 156 | 158 | 173 | 175 | 203 | 203 | 168 | 172 | 177 | 179 | 215 | 215 | 162 | 164 | 174 | 176 |
| LY | LY122 | 156 | 158 | 173 | 175 | 203 | 203 | 168 | 172 | 177 | 179 | 215 | 215 | 160 | 162 | 176 | 180 |
| LY | LY123 | 156 | 158 | 173 | 175 | 203 | 203 | 168 | 172 | 177 | 179 | 215 | 215 | 154 | 162 | 176 | 176 |
| LY | LY124 | 156 | 158 | 173 | 175 | 203 | 203 | 168 | 172 | 177 | 179 | 215 | 215 | 154 | 162 | 176 | 176 |
| LY | LY125 | 156 | 158 | 173 | 175 | 203 | 203 | 168 | 172 | 177 | 179 | 215 | 215 | 154 | 162 | 176 | 176 |
| LY | LY126 | 156 | 158 | 173 | 175 | 203 | 203 | 168 | 172 | 177 | 179 | 215 | 215 | 154 | 162 | 176 | 176 |
| LY | LY127 | 156 | 158 | 173 | 175 | 203 | 203 | 168 | 172 | 177 | 179 | 215 | 215 | 162 | 164 | 174 | 176 |
| LY | LY128 | 156 | 158 | 173 | 175 | 203 | 203 | 168 | 172 | 177 | 179 | 215 | 215 | 160 | 162 | 176 | 180 |
| LY | LY130 | 156 | 158 | 173 | 175 | 203 | 203 | 168 | 172 | 177 | 179 | 215 | 215 | 160 | 162 | 176 | 180 |
| LY | LY131 | 156 | 158 | 173 | 175 | 203 | 203 | 168 | 172 | 177 | 179 | 215 | 215 | 154 | 162 | 176 | 180 |
| LY | LY132 | 156 | 158 | 173 | 175 | 203 | 203 | 168 | 172 | 177 | 179 | 215 | 215 | 154 | 162 | 176 | 176 |
| LY | LY133 | 156 | 158 | 173 | 175 | 203 | 203 | 168 | 172 | 177 | 179 | 215 | 215 | 162 | 164 | 174 | 176 |
| LY | LY135 | 156 | 158 | 173 | 175 | 203 | 206 | 168 | 172 | 177 | 179 | 215 | 215 | 162 | 164 | 174 | 176 |
| LY | LY137 | 156 | 158 | 173 | 175 | 203 | 203 | 168 | 172 | 177 | 179 | 215 | 215 | 154 | 162 | 176 | 176 |
| LY | LY14 | 156 | 158 | 173 | 175 | 203 | 203 | 168 | 172 | 177 | 179 | 215 | 215 | 160 | 162 | 176 | 180 |
| LY | LY140 | 156 | 158 | 173 | 175 | 203 | 203 | 168 | 172 | 177 | 179 | 215 | 215 | 154 | 162 | 176 | 176 |
| LY | LY15 | 156 | 158 | 173 | 175 | 203 | 203 | 168 | 172 | 177 | 179 | 215 | 215 | 160 | 162 | 176 | 180 |
| LY | LY16 | 156 | 158 | 173 | 175 | 203 | 203 | 168 | 172 | 177 | 179 | 215 | 215 | 162 | 164 | 174 | 176 |
| LY | LY19 | 156 | 158 | 173 | 175 | 203 | 203 | 168 | 172 | 177 | 179 | 215 | 215 | 162 | 164 | 174 | 176 |
| LY | LY2 | 156 | 158 | 173 | 175 | 203 | 203 | 168 | 172 | 177 | 179 | 215 | 215 | 154 | 162 | 176 | 176 |
| LY | LY21 | 156 | 158 | 173 | 175 | 203 | 203 | 168 | 172 | 177 | 179 | 215 | 215 | 162 | 164 | 174 | 176 |
| LY | LY22 | 156 | 158 | 173 | 175 | 203 | 203 | 168 | 172 | 177 | 179 | 215 | 215 | 162 | 164 | 174 | 176 |
| LY | LY23 | 156 | 158 | 173 | 175 | 203 | 203 | 168 | 172 | 177 | 179 | 215 | 215 | 162 | 164 | 174 | 176 |

| | | | | | | | | | | | | | | | | | |
|---|---|---|---|---|---|---|---|---|---|---|---|---|---|---|---|---|---|
| LY | LY24 | 156 | 158 | 173 | 175 | 203 | 203 | 168 | 172 | 177 | 179 | 215 | 215 | 160 | 162 | 176 | 180 |
| LY | LY25 | 156 | 158 | 173 | 175 | 203 | 203 | 168 | 172 | 177 | 179 | 215 | 215 | 154 | 162 | 176 | 180 |
| LY | LY27 | 156 | 158 | 173 | 175 | 203 | 203 | 168 | 172 | 177 | 179 | 215 | 215 | 162 | 164 | 174 | 176 |
| LY | LY29 | 156 | 158 | 173 | 175 | 203 | 203 | 168 | 172 | 177 | 179 | 215 | 215 | 162 | 164 | 174 | 176 |
| LY | LY3 | 156 | 158 | 173 | 175 | 203 | 203 | 168 | 172 | 177 | 179 | 215 | 215 | 160 | 162 | 176 | 180 |
| LY | LY31 | 156 | 158 | 173 | 175 | 203 | 203 | 168 | 172 | 177 | 179 | 215 | 215 | 160 | 162 | 176 | 180 |
| LY | LY33 | 156 | 158 | 173 | 175 | 203 | 203 | 168 | 172 | 177 | 179 | 215 | 215 | 154 | 162 | 176 | 180 |
| LY | LY34 | 156 | 158 | 173 | 175 | 203 | 203 | 168 | 172 | 177 | 179 | 215 | 215 | 160 | 162 | 176 | 180 |
| LY | LY35 | 156 | 158 | 173 | 175 | 203 | 203 | 168 | 172 | 177 | 179 | 215 | 215 | 154 | 162 | 176 | 176 |
| LY | LY37 | 156 | 158 | 173 | 175 | 203 | 203 | 168 | 172 | 177 | 179 | 215 | 215 | 154 | 162 | 176 | 176 |
| LY | LY38 | 156 | 158 | 173 | 175 | 203 | 203 | 168 | 172 | 177 | 179 | 215 | 215 | 154 | 162 | 176 | 180 |
| LY | LY39 | 156 | 158 | 173 | 175 | 203 | 203 | 168 | 172 | 177 | 179 | 215 | 215 | 160 | 162 | 176 | 180 |
| LY | LY4 | 156 | 158 | 173 | 175 | 203 | 203 | 168 | 172 | 177 | 179 | 215 | 215 | 154 | 162 | 176 | 180 |
| LY | LY40 | 156 | 158 | 173 | 175 | 203 | 203 | 168 | 172 | 177 | 179 | 215 | 215 | 162 | 164 | 174 | 176 |
| LY | LY41 | 156 | 158 | 173 | 175 | 203 | 203 | 168 | 172 | 177 | 179 | 215 | 215 | 154 | 162 | 176 | 180 |
| LY | LY42 | 156 | 158 | 173 | 175 | 203 | 203 | 168 | 172 | 177 | 179 | 215 | 215 | 162 | 164 | 174 | 176 |
| LY | LY45 | 156 | 158 | 173 | 175 | 203 | 203 | 168 | 172 | 177 | 179 | 215 | 215 | 162 | 164 | 174 | 176 |
| LY | LY46 | 156 | 158 | 173 | 175 | 203 | 203 | 168 | 172 | 177 | 179 | 215 | 215 | 154 | 162 | 176 | 176 |
| LY | LY47 | 156 | 158 | 173 | 175 | 203 | 203 | 168 | 172 | 177 | 179 | 215 | 215 | 154 | 162 | 176 | 176 |
| LY | LY48 | 156 | 158 | 173 | 175 | 203 | 203 | 168 | 172 | 177 | 179 | 215 | 215 | 162 | 164 | 174 | 176 |
| LY | LY50 | 156 | 158 | 173 | 175 | 203 | 203 | 168 | 172 | 177 | 179 | 215 | 215 | 154 | 162 | 176 | 180 |
| LY | LY51 | 156 | 158 | 173 | 175 | 203 | 203 | 168 | 172 | 177 | 179 | 215 | 215 | 160 | 162 | 176 | 180 |
| LY | LY52 | 156 | 158 | 173 | 175 | 203 | 203 | 168 | 172 | 177 | 179 | 215 | 215 | 162 | 164 | 174 | 176 |
| LY | LY54 | 156 | 158 | 173 | 175 | 203 | 203 | 168 | 172 | 177 | 179 | 215 | 215 | 154 | 162 | 176 | 176 |
| LY | LY56 | 156 | 158 | 173 | 175 | 203 | 203 | 168 | 172 | 177 | 179 | 215 | 215 | 154 | 162 | 176 | 176 |
| LY | LY57 | 156 | 158 | 173 | 175 | 203 | 203 | 168 | 172 | 177 | 179 | 215 | 215 | 162 | 164 | 174 | 176 |
| LY | LY58 | 156 | 158 | 173 | 175 | 203 | 203 | 168 | 172 | 177 | 179 | 215 | 215 | 162 | 164 | 174 | 176 |
| LY | LY6 | 156 | 158 | 173 | 175 | 203 | 203 | 168 | 172 | 177 | 179 | 215 | 215 | 154 | 162 | 176 | 180 |
| LY | LY60 | 156 | 158 | 173 | 175 | 203 | 203 | 168 | 172 | 177 | 179 | 215 | 215 | 160 | 162 | 176 | 180 |
| LY | LY61 | 156 | 158 | 173 | 175 | 203 | 203 | 168 | 172 | 177 | 179 | 215 | 215 | 162 | 164 | 174 | 176 |
| LY | LY63 | 156 | 158 | 173 | 175 | 203 | 203 | 168 | 172 | 177 | 179 | 215 | 215 | 154 | 162 | 176 | 176 |

| | | | | | | | | | | | | | | | | | |
|---|---|---|---|---|---|---|---|---|---|---|---|---|---|---|---|---|---|
| LY | LY65 | 156 | 158 | 173 | 175 | 203 | 203 | 168 | 172 | 177 | 179 | 215 | 215 | 154 | 162 | 176 | 176 |
| LY | LY66 | 156 | 158 | 173 | 175 | 203 | 203 | 168 | 172 | 177 | 179 | 215 | 215 | 160 | 162 | 176 | 180 |
| LY | LY68 | 156 | 158 | 173 | 175 | 203 | 203 | 168 | 172 | 177 | 179 | 215 | 215 | 160 | 162 | 176 | 180 |
| LY | LY69 | 156 | 158 | 173 | 175 | 203 | 203 | 168 | 172 | 177 | 179 | 215 | 215 | 154 | 162 | 176 | 180 |
| LY | LY71 | 156 | 158 | 173 | 175 | 203 | 203 | 168 | 172 | 177 | 179 | 215 | 215 | 154 | 162 | 176 | 176 |
| LY | LY72 | 156 | 158 | 173 | 175 | 203 | 206 | 168 | 172 | 177 | 179 | 215 | 215 | 162 | 164 | 174 | 176 |
| LY | LY73 | 156 | 158 | 173 | 175 | 203 | 203 | 168 | 172 | 177 | 179 | 215 | 215 | 154 | 162 | 176 | 180 |
| LY | LY74 | 156 | 158 | 173 | 175 | 203 | 203 | 168 | 172 | 177 | 179 | 215 | 215 | 154 | 162 | 176 | 176 |
| LY | LY75 | 156 | 158 | 173 | 175 | 203 | 203 | 168 | 172 | 177 | 179 | 215 | 215 | 154 | 162 | 176 | 180 |
| LY | LY76 | 156 | 158 | 173 | 175 | 203 | 203 | 168 | 172 | 177 | 179 | 215 | 215 | 154 | 162 | 176 | 180 |
| LY | LY8 | 156 | 158 | 173 | 175 | 203 | 203 | 168 | 172 | 177 | 179 | 215 | 215 | 154 | 162 | 176 | 176 |
| LY | LY80 | 156 | 158 | 173 | 175 | 203 | 203 | 168 | 172 | 177 | 179 | 215 | 215 | 154 | 162 | 176 | 180 |
| LY | LY81 | 156 | 158 | 173 | 175 | 203 | 203 | 168 | 172 | 177 | 179 | 215 | 215 | 154 | 162 | 176 | 176 |
| LY | LY82 | 156 | 158 | 173 | 175 | 203 | 203 | 168 | 172 | 177 | 179 | 215 | 215 | 154 | 162 | 176 | 176 |
| LY | LY83 | 156 | 158 | 173 | 175 | 203 | 203 | 168 | 172 | 177 | 179 | 215 | 215 | 162 | 164 | 174 | 176 |
| LY | LY84 | 156 | 158 | 173 | 175 | 203 | 203 | 168 | 172 | 177 | 179 | 215 | 215 | 162 | 164 | 176 | 180 |
| LY | LY85 | 156 | 158 | 173 | 175 | 203 | 203 | 168 | 172 | 177 | 179 | 215 | 215 | 162 | 164 | 174 | 176 |
| LY | LY86 | 156 | 158 | 173 | 175 | 203 | 203 | 168 | 172 | 177 | 179 | 215 | 215 | 160 | 162 | 176 | 180 |
| LY | LY87 | 156 | 158 | 173 | 175 | 203 | 203 | 168 | 172 | 177 | 179 | 215 | 215 | 154 | 162 | 176 | 176 |
| LY | LY88 | 156 | 158 | 173 | 175 | 203 | 203 | 168 | 172 | 177 | 179 | 215 | 215 | 154 | 162 | 176 | 176 |
| LY | LY9 | 156 | 158 | 173 | 175 | 203 | 203 | 168 | 172 | 177 | 179 | 215 | 215 | 162 | 164 | 174 | 176 |
| LY | LY90 | 156 | 158 | 173 | 175 | 203 | 203 | 168 | 172 | 177 | 179 | 215 | 215 | 154 | 162 | 176 | 176 |
| LY | LY91 | 156 | 158 | 173 | 175 | 203 | 203 | 168 | 172 | 177 | 179 | 215 | 215 | 160 | 162 | 176 | 180 |
| LY | LY92 | 156 | 158 | 173 | 175 | 203 | 203 | 168 | 172 | 177 | 179 | 215 | 215 | 162 | 164 | 174 | 176 |
| LY | LY94 | 156 | 158 | 173 | 175 | 203 | 203 | 168 | 172 | 177 | 179 | 215 | 215 | 154 | 162 | 176 | 180 |
| LY | LY95 | 156 | 158 | 173 | 175 | 203 | 203 | 168 | 172 | 177 | 179 | 215 | 215 | 160 | 162 | 176 | 180 |
| LY | LY96 | 156 | 158 | 173 | 175 | 203 | 203 | 168 | 172 | 177 | 179 | 215 | 215 | 162 | 164 | 174 | 176 |
| LY | LY97 | 156 | 158 | 173 | 175 | 203 | 203 | 168 | 172 | 177 | 179 | 215 | 215 | 154 | 162 | 176 | 176 |
| LY | LY98 | 156 | 158 | 173 | 175 | 203 | 206 | 168 | 172 | 177 | 179 | 215 | 215 | 162 | 164 | 174 | 176 |
| LY | LY99 | 156 | 158 | 173 | 175 | 203 | 203 | 168 | 172 | 177 | 179 | 215 | 215 | 162 | 164 | 174 | 176 |
| QK | QK1 | 160 | 162 | 173 | 175 | 203 | 203 | 168 | 172 | 175 | 177 | 207 | 215 | 162 | 164 | 174 | 176 |

| | | | | | | | | | | | | | | | | | |
|---|---|---|---|---|---|---|---|---|---|---|---|---|---|---|---|---|---|
| QK | QK10 | 160 | 162 | 173 | 175 | 203 | 203 | 168 | 172 | 175 | 177 | 207 | 215 | 162 | 164 | 174 | 176 |
| QK | QK11 | 160 | 162 | 173 | 175 | 203 | 203 | 168 | 172 | 177 | 177 | 207 | 215 | 162 | 164 | 174 | 176 |
| QK | QK12 | 160 | 162 | 173 | 175 | 203 | 203 | 168 | 172 | 177 | 177 | 207 | 215 | 162 | 164 | 174 | 176 |
| QK | QK13 | 160 | 162 | 173 | 175 | 203 | 203 | 168 | 172 | 177 | 177 | 207 | 215 | 162 | 164 | 174 | 176 |
| QK | QK14 | 160 | 162 | 173 | 175 | 203 | 203 | 168 | 172 | 177 | 177 | 207 | 215 | 162 | 164 | 174 | 176 |
| QK | QK15 | 160 | 162 | 173 | 175 | 203 | 203 | 168 | 172 | 177 | 177 | 207 | 215 | 162 | 164 | 174 | 176 |
| QK | QK16 | 160 | 162 | 173 | 175 | 203 | 203 | 168 | 172 | 177 | 177 | 207 | 215 | 162 | 164 | 174 | 176 |
| QK | QK17 | 160 | 162 | 173 | 175 | 203 | 203 | 168 | 172 | 177 | 177 | 207 | 215 | 162 | 164 | 174 | 176 |
| QK | QK18 | 160 | 162 | 173 | 175 | 203 | 203 | 168 | 172 | 177 | 177 | 207 | 215 | 162 | 164 | 174 | 176 |
| QK | QK19 | 160 | 162 | 173 | 175 | 203 | 203 | 168 | 172 | 175 | 177 | 207 | 215 | 162 | 164 | 174 | 176 |
| QK | QK2 | 160 | 162 | 173 | 175 | 203 | 203 | 168 | 172 | 177 | 177 | 207 | 215 | 162 | 164 | 174 | 176 |
| QK | QK20 | 160 | 162 | 173 | 175 | 203 | 203 | 168 | 172 | 177 | 177 | 207 | 215 | 162 | 164 | 174 | 176 |
| QK | QK21 | 160 | 162 | 173 | 175 | 203 | 203 | 168 | 172 | 177 | 177 | 207 | 215 | 162 | 164 | 174 | 176 |
| QK | QK22 | 160 | 162 | 173 | 175 | 203 | 203 | 168 | 172 | 175 | 177 | 207 | 215 | 162 | 164 | 174 | 176 |
| QK | QK23 | 160 | 162 | 173 | 175 | 203 | 203 | 168 | 172 | 177 | 177 | 207 | 215 | 162 | 164 | 174 | 176 |
| QK | QK24 | 160 | 162 | 173 | 175 | 203 | 203 | 168 | 172 | 177 | 177 | 207 | 215 | 162 | 164 | 174 | 176 |
| QK | QK25 | 160 | 162 | 173 | 175 | 203 | 203 | 168 | 172 | 177 | 177 | 207 | 215 | 162 | 164 | 174 | 176 |
| QK | QK26 | 160 | 162 | 173 | 175 | 203 | 203 | 168 | 172 | 177 | 177 | 207 | 215 | 162 | 164 | 174 | 176 |
| QK | QK27 | 160 | 162 | 173 | 175 | 203 | 203 | 168 | 172 | 177 | 177 | 207 | 215 | 162 | 164 | 174 | 176 |
| QK | QK28 | 160 | 162 | 173 | 175 | 203 | 203 | 168 | 172 | 177 | 177 | 207 | 215 | 162 | 164 | 174 | 176 |
| QK | QK29 | 160 | 162 | 173 | 175 | 203 | 203 | 168 | 172 | 177 | 177 | 207 | 215 | 162 | 164 | 174 | 176 |
| QK | QK3 | 160 | 162 | 173 | 175 | 203 | 203 | 168 | 172 | 177 | 177 | 207 | 215 | 162 | 164 | 174 | 176 |
| QK | QK30 | 160 | 162 | 173 | 175 | 203 | 203 | 168 | 172 | 175 | 177 | 207 | 215 | 162 | 164 | 174 | 176 |
| QK | QK31 | 160 | 162 | 173 | 175 | 203 | 203 | 168 | 172 | 177 | 177 | 207 | 215 | 162 | 164 | 174 | 176 |
| QK | QK32 | 160 | 162 | 173 | 175 | 203 | 203 | 168 | 172 | 177 | 177 | 207 | 215 | 162 | 164 | 174 | 176 |
| QK | QK33 | 160 | 162 | 173 | 175 | 203 | 203 | 168 | 172 | 177 | 177 | 207 | 215 | 162 | 164 | 174 | 176 |
| QK | QK35 | 160 | 162 | 173 | 175 | 203 | 203 | 168 | 172 | 177 | 177 | 207 | 215 | 162 | 164 | 174 | 176 |
| QK | QK37 | 160 | 162 | 173 | 175 | 203 | 203 | 168 | 172 | 177 | 177 | 207 | 215 | 162 | 164 | 174 | 176 |
| QK | QK38 | 160 | 162 | 173 | 175 | 203 | 203 | 168 | 172 | 177 | 177 | 207 | 215 | 162 | 164 | 174 | 176 |
| QK | QK39 | 160 | 162 | 173 | 175 | 203 | 203 | 168 | 172 | 177 | 177 | 207 | 215 | 162 | 164 | 174 | 176 |
| QK | QK4 | 160 | 162 | 173 | 175 | 203 | 203 | 168 | 172 | 175 | 177 | 207 | 215 | 162 | 164 | 174 | 176 |

| | | | | | | | | | | | | | | | | | |
|---|---|---|---|---|---|---|---|---|---|---|---|---|---|---|---|---|---|
| QK | QK40 | 160 | 162 | 173 | 175 | 203 | 203 | 168 | 172 | 175 | 177 | 207 | 215 | 162 | 164 | 174 | 176 |
| QK | QK41 | 160 | 162 | 173 | 175 | 203 | 203 | 168 | 172 | 177 | 177 | 207 | 215 | 162 | 164 | 174 | 176 |
| QK | QK42 | 160 | 162 | 173 | 175 | 203 | 203 | 168 | 172 | 177 | 177 | 207 | 215 | 162 | 164 | 174 | 176 |
| QK | QK43 | 160 | 162 | 173 | 175 | 203 | 203 | 168 | 172 | 177 | 177 | 207 | 215 | 162 | 164 | 174 | 176 |
| QK | QK44 | 160 | 162 | 173 | 175 | 203 | 203 | 168 | 172 | 175 | 177 | 207 | 215 | 162 | 164 | 174 | 176 |
| QK | QK45 | 160 | 162 | 173 | 175 | 203 | 203 | 168 | 172 | 177 | 177 | 207 | 215 | 162 | 164 | 174 | 176 |
| QK | QK46 | 160 | 162 | 173 | 175 | 203 | 203 | 168 | 172 | 177 | 177 | 207 | 215 | 162 | 164 | 174 | 176 |
| QK | QK47 | 160 | 162 | 173 | 175 | 203 | 203 | 168 | 172 | 177 | 177 | 207 | 215 | 162 | 164 | 174 | 176 |
| QK | QK48 | 160 | 162 | 173 | 175 | 203 | 203 | 168 | 172 | 175 | 177 | 207 | 215 | 162 | 164 | 174 | 176 |
| QK | QK49 | 160 | 162 | 173 | 175 | 203 | 203 | 168 | 172 | 177 | 177 | 207 | 215 | 162 | 164 | 174 | 176 |
| QK | QK5 | 160 | 162 | 173 | 175 | 203 | 203 | 168 | 172 | 177 | 177 | 207 | 215 | 162 | 164 | 174 | 176 |
| QK | QK50 | 160 | 162 | 173 | 175 | 203 | 203 | 168 | 172 | 177 | 177 | 207 | 215 | 162 | 164 | 174 | 176 |
| QK | QK51 | 160 | 162 | 173 | 175 | 203 | 203 | 168 | 172 | 177 | 177 | 207 | 215 | 162 | 164 | 174 | 176 |
| QK | QK52 | 160 | 162 | 173 | 175 | 203 | 203 | 168 | 172 | 175 | 177 | 207 | 215 | 162 | 164 | 174 | 176 |
| QK | QK53 | 160 | 162 | 173 | 175 | 203 | 203 | 168 | 172 | 177 | 177 | 207 | 215 | 162 | 164 | 174 | 176 |
| QK | QK54 | 160 | 162 | 173 | 175 | 203 | 203 | 168 | 172 | 177 | 177 | 207 | 215 | 162 | 164 | 174 | 176 |
| QK | QK55 | 160 | 162 | 173 | 175 | 203 | 203 | 168 | 172 | 177 | 177 | 207 | 215 | 162 | 164 | 174 | 176 |
| QK | QK57 | 160 | 162 | 173 | 175 | 203 | 203 | 168 | 172 | 177 | 177 | 207 | 215 | 162 | 164 | 174 | 176 |
| QK | QK58 | 160 | 162 | 173 | 175 | 203 | 203 | 168 | 172 | 177 | 177 | 207 | 215 | 162 | 164 | 174 | 176 |
| QK | QK59 | 160 | 162 | 173 | 175 | 203 | 203 | 168 | 172 | 175 | 177 | 207 | 215 | 162 | 164 | 174 | 176 |
| QK | QK6 | 160 | 162 | 173 | 175 | 203 | 203 | 168 | 172 | 177 | 177 | 207 | 215 | 162 | 164 | 174 | 176 |
| QK | QK60 | 160 | 162 | 173 | 175 | 203 | 203 | 168 | 172 | 177 | 177 | 207 | 215 | 162 | 164 | 174 | 176 |
| QK | QK62 | 160 | 162 | 173 | 175 | 203 | 203 | 168 | 172 | 177 | 177 | 207 | 215 | 162 | 164 | 174 | 176 |
| QK | QK63 | 160 | 162 | 173 | 175 | 203 | 203 | 168 | 172 | 177 | 177 | 207 | 215 | 162 | 164 | 174 | 176 |
| QK | QK64 | 160 | 162 | 173 | 175 | 203 | 203 | 168 | 172 | 177 | 177 | 207 | 215 | 162 | 164 | 174 | 176 |
| QK | QK65 | 160 | 162 | 173 | 175 | 203 | 203 | 168 | 172 | 177 | 177 | 207 | 215 | 162 | 164 | 174 | 176 |
| QK | QK66 | 160 | 162 | 173 | 175 | 203 | 203 | 168 | 172 | 175 | 177 | 207 | 215 | 162 | 164 | 174 | 176 |
| QK | QK67 | 160 | 162 | 173 | 175 | 203 | 203 | 168 | 172 | 177 | 177 | 207 | 215 | 162 | 164 | 174 | 176 |
| QK | QK68 | 160 | 162 | 173 | 175 | 203 | 203 | 168 | 172 | 177 | 177 | 207 | 215 | 162 | 164 | 174 | 176 |
| QK | QK7 | 160 | 162 | 173 | 175 | 203 | 203 | 168 | 172 | 177 | 177 | 207 | 215 | 162 | 164 | 174 | 176 |
| QK | QK70 | 160 | 162 | 173 | 175 | 203 | 203 | 168 | 172 | 177 | 177 | 207 | 215 | 162 | 164 | 174 | 176 |

| | | | | | | | | | | | | | | | | | | | |
|---|---|---|---|---|---|---|---|---|---|---|---|---|---|---|---|---|---|---|---|
| QK | QK71 | 160 | 162 | 173 | 175 | 203 | 203 | 168 | 172 | 177 | 177 | 207 | 215 | 162 | 164 | 174 | 176 |
| QK | QK72 | 160 | 162 | 173 | 175 | 203 | 203 | 168 | 172 | 177 | 177 | 207 | 215 | 162 | 164 | 174 | 176 |
| QK | QK73 | 160 | 162 | 173 | 175 | 203 | 203 | 168 | 172 | 177 | 177 | 207 | 215 | 162 | 164 | 174 | 176 |
| QK | QK74 | 160 | 162 | 173 | 175 | 203 | 203 | 168 | 172 | 177 | 177 | 207 | 215 | 162 | 164 | 174 | 176 |
| QK | QK75 | 160 | 162 | 173 | 175 | 203 | 203 | 168 | 172 | 177 | 177 | 207 | 215 | 162 | 164 | 174 | 176 |
| QK | QK76 | 160 | 162 | 173 | 175 | 203 | 203 | 168 | 172 | 177 | 177 | 207 | 215 | 162 | 164 | 174 | 176 |
| QK | QK77 | 160 | 162 | 173 | 175 | 203 | 203 | 168 | 172 | 177 | 177 | 207 | 215 | 162 | 164 | 174 | 176 |
| QK | QK78 | 160 | 162 | 173 | 175 | 203 | 203 | 168 | 172 | 177 | 177 | 207 | 215 | 162 | 164 | 174 | 176 |
| QK | QK80 | 160 | 162 | 173 | 175 | 203 | 203 | 168 | 172 | 175 | 177 | 207 | 215 | 162 | 164 | 174 | 176 |
| QK | QK81 | 160 | 162 | 173 | 175 | 203 | 203 | 168 | 172 | 177 | 177 | 207 | 215 | 162 | 164 | 174 | 176 |
| QK | QK82 | 160 | 162 | 173 | 175 | 203 | 203 | 168 | 172 | 175 | 177 | 207 | 215 | 162 | 164 | 174 | 176 |
| QK | QK9 | 160 | 162 | 173 | 175 | 203 | 203 | 168 | 172 | 177 | 177 | 207 | 215 | 162 | 164 | 174 | 176 |
| CL | F01 | 156 | 158 | 175 | 175 | 203 | 206 | 168 | 172 | 177 | 179 | 207 | 207 | 162 | 164 | 174 | 176 |
| CL | F03 | 156 | 156 | 175 | 175 | 203 | 206 | 168 | 172 | 177 | 179 | 207 | 219 | 162 | 164 | 174 | 176 |
| CL | F04 | 156 | 158 | 175 | 175 | 203 | 206 | 168 | 172 | 177 | 179 | 207 | 219 | 162 | 164 | 176 | 172 |
| CL | F08 | 156 | 158 | 175 | 175 | 203 | 206 | 168 | 172 | 177 | 179 | 207 | 219 | 162 | 164 | 176 | 176 |
| CL | F11 | 156 | 158 | 175 | 175 | 203 | 206 | 168 | 172 | 177 | 179 | 207 | 219 | 162 | 164 | 174 | 176 |
| CL | F12 | 156 | 158 | 175 | 175 | 203 | 206 | 168 | 172 | 177 | 179 | 207 | 219 | 162 | 164 | 174 | 176 |
| CL | F13 | 156 | 158 | 175 | 175 | 203 | 206 | 168 | 172 | 177 | 179 | 207 | 219 | 162 | 164 | 174 | 176 |
| CL | F17 | 156 | 158 | 175 | 175 | 203 | 206 | 168 | 172 | 177 | 179 | 207 | 219 | 162 | 164 | 174 | 176 |
| CL | F18 | 156 | 158 | 175 | 175 | 203 | 206 | 168 | 172 | 177 | 179 | 207 | 219 | 162 | 164 | 176 | 172 |
| CL | F19 | 156 | 158 | 175 | 175 | 203 | 206 | 168 | 172 | 177 | 179 | 207 | 219 | 162 | 164 | 174 | 176 |
| CL | F20 | 156 | 158 | 175 | 175 | 203 | 206 | 168 | 172 | 177 | 179 | 207 | 219 | 162 | 164 | 174 | 176 |
| CL | F21 | 156 | 158 | 175 | 175 | 203 | 206 | 168 | 172 | 177 | 179 | 207 | 219 | 162 | 164 | 174 | 176 |
| CL | F24 | 156 | 158 | 175 | 175 | 203 | 206 | 168 | 172 | 177 | 179 | 207 | 219 | 162 | 164 | 174 | 176 |
| CL | F25 | 156 | 158 | 175 | 175 | 203 | 206 | 168 | 172 | 177 | 179 | 207 | 219 | 162 | 164 | 174 | 176 |
| CL | F26 | 156 | 158 | 175 | 175 | 203 | 206 | 168 | 172 | 177 | 179 | 207 | 219 | 162 | 164 | 174 | 176 |
| CL | F27 | 156 | 158 | 175 | 175 | 203 | 206 | 168 | 172 | 177 | 179 | 207 | 219 | 162 | 164 | 174 | 176 |
| CL | F28 | 156 | 158 | 175 | 175 | 203 | 206 | 168 | 172 | 177 | 179 | 207 | 219 | 162 | 164 | 174 | 176 |
| CL | F30 | 156 | 158 | 175 | 175 | 203 | 206 | 168 | 172 | 177 | 179 | 207 | 219 | 162 | 164 | 174 | 176 |
| CL | F31 | 156 | 158 | 175 | 175 | 203 | 206 | 168 | 172 | 177 | 179 | 207 | 219 | 162 | 164 | 174 | 176 |

| | | | | | | | | | | | | | | | | | |
|---|---|---|---|---|---|---|---|---|---|---|---|---|---|---|---|---|---|
| CL | F32 | 156 | 158 | 173 | 173 | 203 | 206 | 168 | 172 | 177 | 179 | 207 | 219 | 162 | 164 | 174 | 176 |
| CL | F34 | 156 | 158 | 175 | 175 | 203 | 206 | 168 | 172 | 177 | 179 | 207 | 219 | 162 | 164 | 174 | 176 |
| CL | F35 | 156 | 158 | 175 | 175 | 203 | 206 | 168 | 172 | 177 | 179 | 207 | 219 | 162 | 164 | 174 | 176 |
| FZ | F069 | 158 | 158 | 173 | 175 | 203 | 203 | 168 | 172 | 177 | 179 | 215 | 213 | 160 | 162 | 176 | 176 |
| FZ | F070 | 158 | 158 | 173 | 175 | 203 | 203 | 168 | 172 | 177 | 179 | 215 | 213 | 162 | 162 | 176 | 176 |
| FZ | F071 | 156 | 158 | 173 | 175 | 203 | 203 | 168 | 172 | 177 | 179 | 215 | 213 | 160 | 162 | 176 | 176 |
| FZ | F100 | 156 | 158 | 173 | 175 | 203 | 203 | 168 | 172 | 177 | 179 | 215 | 213 | 162 | 164 | 176 | 172 |
| FZ | F101 | 156 | 158 | 173 | 175 | 203 | 203 | 168 | 172 | 177 | 179 | 215 | 213 | 162 | 164 | 174 | 176 |
| FZ | F102 | 156 | 158 | 173 | 175 | 203 | 203 | 168 | 172 | 177 | 179 | 215 | 213 | 162 | 164 | 176 | 176 |
| FZ | F103 | 156 | 158 | 173 | 175 | 203 | 203 | 168 | 172 | 177 | 179 | 215 | 213 | 162 | 164 | 176 | 176 |
| FZ | F104 | 156 | 158 | 173 | 175 | 203 | 203 | 168 | 172 | 177 | 179 | 215 | 213 | 162 | 164 | 176 | 176 |
| FZ | F105 | 156 | 158 | 173 | 175 | 203 | 203 | 168 | 172 | 177 | 179 | 215 | 213 | 162 | 164 | 174 | 176 |
| FZ | F106 | 134 | 158 | 173 | 175 | 203 | 203 | 168 | 170 | 177 | 177 | 215 | 213 | 162 | 164 | 174 | 176 |
| FZ | F107 | 134 | 158 | 173 | 175 | 203 | 203 | 166 | 170 | 177 | 177 | 215 | 213 | 162 | 164 | 174 | 176 |
| FZ | F68 | 156 | 158 | 173 | 175 | 203 | 203 | 168 | 172 | 177 | 179 | 215 | 213 | 162 | 164 | 174 | 176 |
| FZ | F73 | 156 | 158 | 173 | 175 | 203 | 203 | 168 | 172 | 177 | 179 | 215 | 213 | 162 | 164 | 174 | 176 |
| FZ | F74 | 156 | 158 | 173 | 175 | 203 | 203 | 168 | 172 | 177 | 179 | 215 | 213 | 162 | 164 | 176 | 176 |
| FZ | F75 | 156 | 158 | 173 | 175 | 203 | 203 | 168 | 172 | 177 | 179 | 215 | 213 | 162 | 164 | 176 | 176 |
| FZ | F77 | 156 | 158 | 173 | 175 | 203 | 203 | 168 | 172 | 177 | 179 | 215 | 213 | 162 | 164 | 176 | 172 |
| FZ | F78 | 156 | 158 | 173 | 175 | 203 | 203 | 168 | 172 | 177 | 179 | 215 | 213 | 162 | 164 | 174 | 176 |
| FZ | F79 | 156 | 158 | 173 | 175 | 203 | 203 | 168 | 172 | 177 | 179 | 215 | 213 | 162 | 164 | 174 | 176 |
| FZ | F80 | 156 | 158 | 173 | 175 | 203 | 203 | 168 | 172 | 177 | 179 | 215 | 213 | 162 | 164 | 176 | 176 |
| FZ | F81 | 156 | 158 | 173 | 175 | 203 | 203 | 168 | 172 | 177 | 179 | 215 | 213 | 162 | 164 | 174 | 176 |
| FZ | F82 | 156 | 158 | 173 | 175 | 203 | 203 | 168 | 172 | 177 | 179 | 215 | 215 | 162 | 164 | 174 | 176 |
| FZ | F83 | 156 | 158 | 173 | 175 | 203 | 203 | 168 | 172 | 177 | 179 | 215 | 213 | 162 | 164 | 174 | 172 |
| FZ | F84 | 156 | 158 | 173 | 175 | 203 | 203 | 168 | 172 | 177 | 179 | 215 | 213 | 162 | 164 | 174 | 176 |
| FZ | F85 | 156 | 158 | 173 | 175 | 203 | 203 | 168 | 172 | 177 | 179 | 215 | 213 | 162 | 164 | 174 | 176 |
| FZ | F86 | 156 | 158 | 173 | 175 | 203 | 203 | 168 | 172 | 177 | 179 | 215 | 213 | 162 | 164 | 174 | 176 |
| FZ | F87 | 156 | 158 | 173 | 175 | 203 | 203 | 168 | 172 | 177 | 179 | 215 | 213 | 162 | 164 | 174 | 176 |
| FZ | F88 | 156 | 158 | 173 | 175 | 203 | 203 | 168 | 172 | 177 | 179 | 215 | 213 | 162 | 164 | 174 | 176 |
| FZ | F89 | 156 | 158 | 173 | 175 | 203 | 203 | 168 | 172 | 177 | 179 | 215 | 213 | 162 | 164 | 174 | 176 |

| | | | | | | | | | | | | | | | | | |
|---|---|---|---|---|---|---|---|---|---|---|---|---|---|---|---|---|---|
| FZ | F90 | 156 | 158 | 173 | 175 | 203 | 203 | 168 | 172 | 177 | 179 | 215 | 213 | 162 | 164 | 174 | 176 |
| FZ | F91 | 156 | 158 | 173 | 175 | 203 | 203 | 168 | 172 | 177 | 179 | 215 | 213 | 162 | 164 | 174 | 176 |
| FZ | F92 | 156 | 158 | 173 | 175 | 203 | 203 | 168 | 172 | 177 | 179 | 215 | 213 | 162 | 164 | 174 | 176 |
| FZ | F93 | 156 | 158 | 173 | 175 | 203 | 203 | 168 | 172 | 177 | 179 | 215 | 213 | 162 | 164 | 174 | 176 |
| FZ | F94 | 156 | 158 | 173 | 175 | 203 | 203 | 168 | 172 | 177 | 179 | 215 | 213 | 162 | 164 | 174 | 176 |
| FZ | F95 | 156 | 158 | 173 | 175 | 203 | 203 | 168 | 172 | 177 | 179 | 215 | 213 | 162 | 164 | 174 | 176 |
| FZ | F96 | 156 | 158 | 173 | 175 | 203 | 203 | 168 | 172 | 177 | 179 | 215 | 213 | 162 | 164 | 174 | 176 |
| FZ | F97 | 156 | 158 | 173 | 175 | 203 | 203 | 168 | 172 | 177 | 179 | 215 | 213 | 162 | 164 | 174 | 176 |
| FZ | F98 | 156 | 158 | 173 | 173 | 203 | 203 | 168 | 172 | 177 | 179 | 215 | 213 | 162 | 164 | 174 | 176 |
| FZ | F99 | 156 | 158 | 173 | 175 | 203 | 203 | 168 | 172 | 177 | 179 | 215 | 213 | 162 | 164 | 174 | 176 |
